# Intelligent Integrated System for Fruit Detection Using Multi-UAV Imaging and Deep Learning †

**DOI:** 10.3390/s24061913

**Published:** 2024-03-16

**Authors:** Oleksandr Melnychenko, Lukasz Scislo, Oleg Savenko, Anatoliy Sachenko, Pavlo Radiuk

**Affiliations:** 1 Faculty of Information Technologies, Khmelnytskyi National University, 11, Instytuts’ka Str., 29016 Khmelnytskyi, Ukraine; oleksandr.melnychenko@live.com (O.M.); savenko_oleg_st@ukr.net (O.S.); radiukp@khmnu.edu.ua (P.R.); 2 Faculty of Electrical and Computer Engineering, Cracow University of Technology, Warszawska 24, 31-155 Craków, Poland; lscislo@pk.edu.pl; 3 Department of Informatics and Teleinformatics, Kazimierz Pulaski University of Technology and Humanities in Radom, ul. Malczewskiego 29, 26-600 Radom, Poland; 4 Research Institute for Intelligent Computer Systems, West Ukrainian National University, 11, Lvivska Str., 46009 Ternopil, Ukraine

**Keywords:** fruit detection, fruit yield estimation, deep learning, YOLOv5, unmanned aerial vehicle, video stream transmission, synchronization and autonomous movement

## Abstract

In the context of Industry 4.0, one of the most significant challenges is enhancing efficiency in sectors like agriculture by using intelligent sensors and advanced computing. Specifically, the task of fruit detection and counting in orchards represents a complex issue that is crucial for efficient orchard management and harvest preparation. Traditional techniques often fail to provide the timely and precise data necessary for these tasks. With the agricultural sector increasingly relying on technological advancements, the integration of innovative solutions is essential. This study presents a novel approach that combines artificial intelligence (AI), deep learning (DL), and unmanned aerial vehicles (UAVs). The proposed approach demonstrates superior real-time capabilities in fruit detection and counting, utilizing a combination of AI techniques and multi-UAV systems. The core innovation of this approach is its ability to simultaneously capture and synchronize video frames from multiple UAV cameras, converting them into a cohesive data structure and, ultimately, a continuous image. This integration is further enhanced by image quality optimization techniques, ensuring the high-resolution and accurate detection of targeted objects during UAV operations. Its effectiveness is proven by experiments, achieving a high mean average precision rate of 86.8% in fruit detection and counting, which surpasses existing technologies. Additionally, it maintains low average error rates, with a false positive rate at 14.7% and a false negative rate at 18.3%, even under challenging weather conditions like cloudiness. Overall, the practical implications of this multi-UAV imaging and DL-based approach are vast, particularly for real-time fruit recognition in orchards, marking a significant stride forward in the realm of digital agriculture that aligns with the objectives of Industry 4.0.

## 1. Introduction

In the era of Industry 4.0, the blend of smart sensors and advanced computing is transforming various industries, especially agriculture, which is a critical sector for global food security. This field of industry faces significant challenges such as changing climates [1,2], diminishing resources [3], and rising production costs [4], further compounded by social-economic and geopolitical tensions [1,3]. These factors call for creative solutions to maintain both ecological balance and economic viability [2,4,5], especially given the growing world population and shifting climate conditions [6,7]. The integration of cutting-edge technologies is crucial for ensuring consistent food production amidst these challenges [8].

In this scenario, using unmanned aerial vehicles (UAVs) [9] together with deep learning (DL) [10,11,12] represents an innovative solution in precision agriculture [13], especially for identifying fruits in orchards [14,15]. However, this combination comes with its own set of complexities. Gathering real-time data in orchard settings encounters several challenges [13,16]. The varied characteristics of fruits (such as shape, size, color, and texture) and the ever-changing environmental conditions (like lighting conditions and weather) create substantial obstacles to data collection and analysis [17,18,19]. Additionally, accuracy in detecting and categorizing fruits relies heavily on high-quality data, requiring careful image capture and preprocessing [20]. Traditional methods using a single drone often fail to gather enough data, due to restrictions like battery life, carrying capacity, and limited viewing angles [21,22].

To overcome the abovementioned issues, employing multiple UAVs is seen as a robust strategy. Operating several UAVs working together allows for broader coverage [20,23], varied data collection angles [24], and a safety net to prevent data loss [25], ensuring the thorough monitoring of orchards [26]. While capturing diverse viewpoints through successive flights of a single drone could be considered adequate [22], this approach does not match the extensive and multi-dimensional data collection achievable through the simultaneous operation of multiple drones [27,28]. Based on these insights [17,23,24,25,26,27,28], the collaborative work of multiple drones provides broader coverage, diverse data collection perspectives, and a safety net against data loss, making it a compelling choice for advancing fruit detection and counting techniques. To conclude, deploying multiple UAVs seems a more feasible and comprehensive approach than relying on a single drone across flight missions in varied environmental conditions.

Merging multiple UAVs, computer vision, and DL in fruit detection fits perfectly with the vision of Industry 4.0 [29]. It embodies the fusion of advanced sensors, autonomous systems, and smart data analysis, thereby significantly enhancing intelligent agriculture [26,30]. This framework takes advantage of the capabilities of DL, particularly convolutional neural networks (CNNs) and deep convolutional neural networks (DCNNs) [31], for intricate image analysis and object recognition [19,32]. These technologies are not just exceptional at identifying fruit features against complex orchard backgrounds but are also crucial for achieving the computational efficiency and flexibility needed for real-time, in-field applications [28,33].

DCNNs have recently shown remarkable success in the classification and identification of structural objects. Significant contributions have been made regarding fruit detection using DCNNs, yet they do have some limitations. For instance, Mai et al. [34] introduced a Faster R-CNN model for apple detection in orchards, achieving high detection rates but potentially overfitting to specific orchard and apple types alongside high computational demands. Chu et al. [35] advanced this with a Suppression Mask R-CNN, enhancing robustness in complex orchards and achieving an impressive F_1_-score of 90.5%, although its adaptability to various apple types and orchards may require further adaptation. Biffi et al. [36] employed an adaptive training sample selection technique with ResNet50 and FPN, which excelled in dense orchards but showed vulnerability in adverse weather conditions and with varied apple types. Lastly, Sun et al. [37] proposed a lightweight YOLOv5-CS that reduced detection speed by 15.56% (in floating-point operations) and increased average precision to 99.1% for real-time applications. However, the real-world applicability of YOLOv5-CS in diverse orchard conditions and its performance with small or overlapping fruits warrant further exploration. To summarize, each work marked a progression in the field, addressing previous limitations while also introducing new challenges that must be addressed.

Overall, there have been remarkable advancements in intelligent sensors and advanced computing that profoundly impact diverse sectors, including precision agriculture. One of the key challenges in this domain is the development of sophisticated systems for automated fruit detection in orchard environments [18,38]. Traditional models, such as Faster R-CNN and Suppression Mask R-CNN, highlight the need for a detection system that excels across various fruit varieties and orchard configurations and offers computational efficiency for real-time applications. This is particularly crucial for fruit detection using multiple UAVs and mobile platforms, where resource limitations and stringent operational requirements are prevalent. Existing approaches often fall short in terms of generalization, real-world applicability, and performance under varying environmental conditions. There is a compelling need for innovative detection methods that utilize multi-UAV imaging for extensive data collection, coupled with the computational prowess of models like YOLOv5. Such systems could significantly facilitate automated fruit harvesting with great efficiency, yielding high detection accuracy, minimal computational load, and adaptability to diverse environmental conditions and fruit types.

Thus, the goal of this study is to improve the efficiency of fruit detection and counting in orchard settings. This advancement is aimed at facilitating the process of real-time fruit harvest monitoring by leveraging multiple UAVs for the acquisition of high-quality photographic data. The scientific contributions of this work include: (i) designing a new method for the dynamic capture of images obtained from multiple UAVs, ensuring synchronization between different UAVs and autonomous movement of the entire UAV group, from the designated initial point to the final mission points; (ii) designing a new method for real-time video stream synchronization to ensure timely results and detect structural objects missed in previous missions; (iii) introducing a novel DCNN architecture called YOLOv5-v1 for detecting specified structural objects in images, which improves detection accuracy and reduces its training time; (iv) improving the method for counting the specified structural objects from obtained images using a group of UAVs.

The rest of the paper is organized as follows. Section 2 introduces the methods and approaches employed for data collection and processing, along with the various algorithms used in this research. It also discusses the formation of video data acquired by the UAV group in the target zone, detailing the process of video data acquisition and transmission. Section 3 presents the experimental results and Section 4 discusses them. Lastly, Section 5 summarizes the study and suggests potential areas for future research.

## 2. Materials and Methods

**Figure 1:**
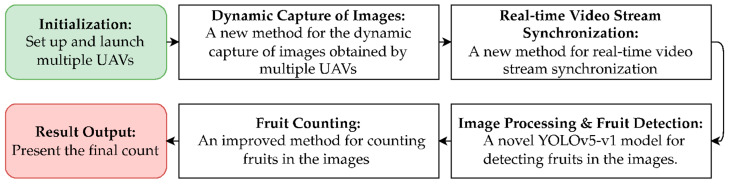
The flowchart outlines the proposed methodological flow for UAV-based fruit counting, which includes the setup and launch of UAVs, dynamic image capture, real-time video stream synchronization, and a novel YOLOv5-v1 model for image processing and fruit detection.

The proposed approach was designed to improve the efficiency of fruit detection and counting in an orchard using multiple UAVs. It includes (i) a new method for the dynamic capture of images obtained from multiple UAVs; (ii) a new method for real-time video stream synchronization to ensure timely results and detect structural objects missed in previous missions; (iii) a method for detecting the specified structural objects (fruits) in images based on the proposed DCNN architecture, which is called YOLOv5-v1; (iv) an improved method for counting the specified structural objects from obtained images using a group of UAVs. The proposed approach is illustrated in Figure 1.

In the following sections, we describe each part of the proposed approach as a novel or improved method.

### 2.1. Method for the Dynamic Capture of Specified Structural Objects

**Figure 2:**
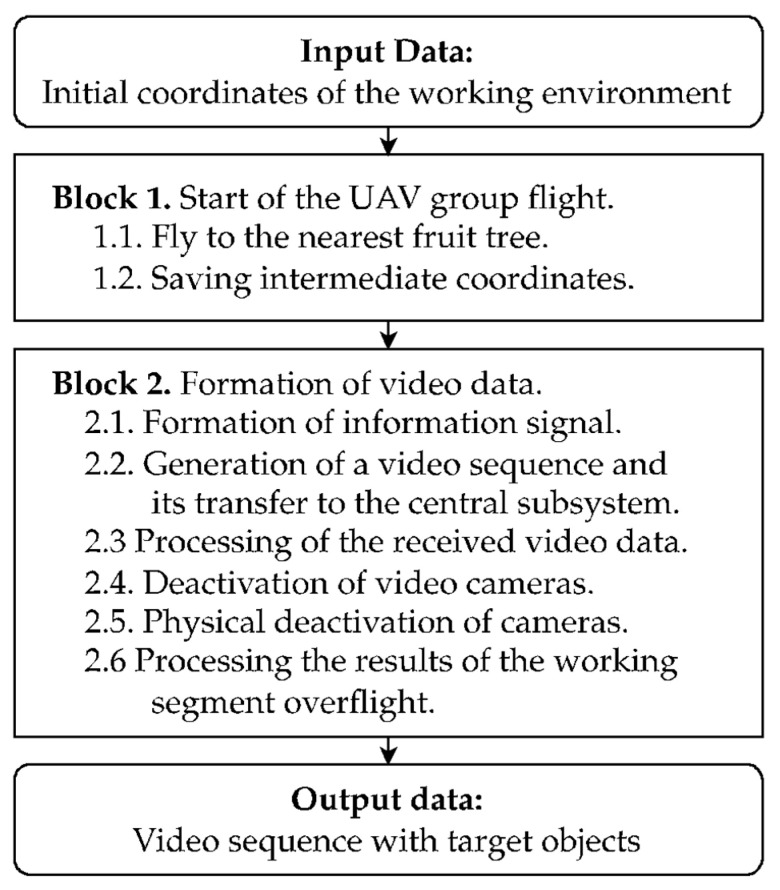
The scheme illustrates the proposed method for the dynamic capture of specified structural objects using UAV technology. The initial phase involves deploying a UAV group to the designated coordinates and capturing intermediate locations. The subsequent phase focuses on video data handling, including signal formation, video sequence generation, transmission to the central unit, data processing, and camera deactivation. The method ends in a video sequence featuring targeted objects, indicating successful data acquisition by a group of UAVs.

The proposed method for the dynamic capture of specified structural objects using a group of UAVs is illustrated by a set of steps (Figure 2).

The input data comprise the following dataset:

- Coordinates of the starting point in the working environment;
- Coordinates of the ending point in the working environment;
- Matrix of coordinates for the initial points of the work segments;
- Matrix of coordinates for the end points of the work segments;
- Matrix of coordinates for the initial UAV trajectory.

The method for dynamically capturing images of structural objects of a similar nature in a three-dimensional space (as depicted in Figure 2) involves the step-by-step execution of several blocks. A detailed description of this method is provided in Appendix A.

### 2.2. Method for Synchronizing Video Streams in Real Time

**Figure 3:**
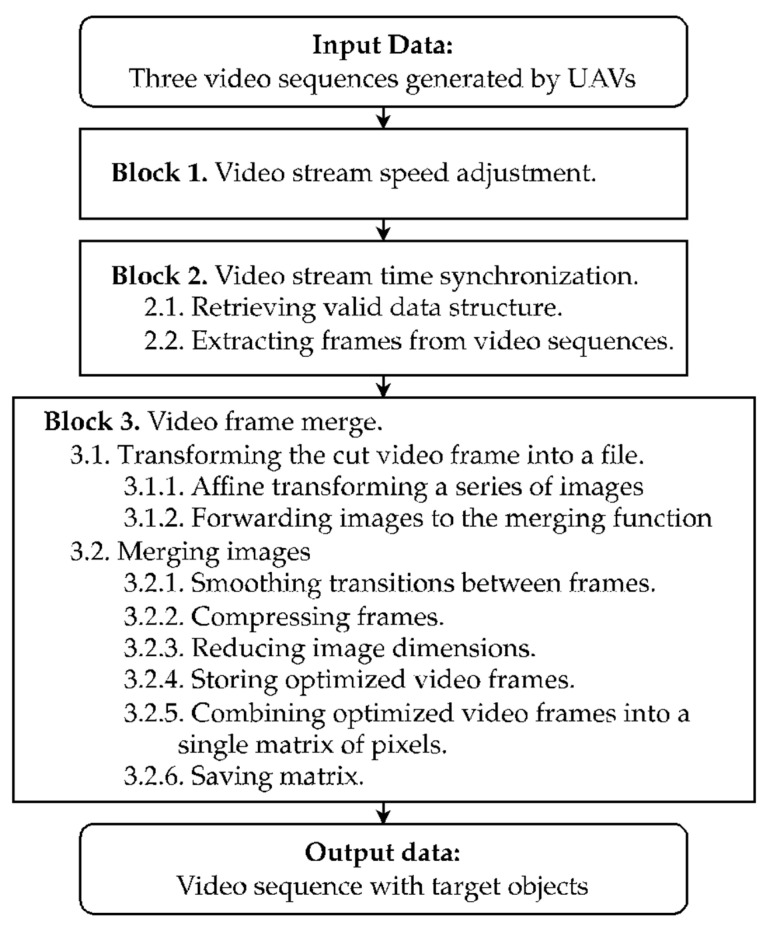
The scheme of the proposed method for synchronizing video streams from multiple UAVs in real time. The blocks of the method include the speed adjustment of video streams, temporal synchronization, and subsequent merging of video frames. These steps are depicted as sequential blocks leading to the final output—a video sequence highlighting target structural objects for detection and counting. This flowchart encapsulates the systematic transformation of raw UAV footage into analyzable data, which is crucial for precise fruit quantification in orchard management.

Synchronizing video streams from various UAVs can be a complex task, one that is influenced by numerous factors. For example, the UAVs may be equipped with different camera models, the pace at which videos are recorded might vary, and there may be distortions in the video sequences or instances where they are not received at all. Additionally, the flight dynamics and video capturing capabilities can greatly vary between UAVs from different manufacturers, potentially leading to decreased detection quality and inaccuracies in identifying the intended structural objects. To address these challenges, we propose a novel method for the real-time synchronization of video streams (Figure 3).

The method illustrated in Figure 3 involves compiling the video sequences captured by each UAV during a mission and seamlessly integrating the video frames into a consolidated image of the fruit tree.

The proposed method for synchronizing video streams in real time is described in detail in Appendix B.

### 2.3. Methods and Means for Detecting, Tracking, and Counting the Specified Structural Objects

#### 2.3.1. Method for Detecting the Specified Structural Objects

The task of automatically identifying fruits on orchard trees takes place within a complex lattice-like setting, wherein the trees bearing fruits are systematically aligned in rows. When a UAV is deployed for a mission, it may capture images of fruits situated in the foreground or background of the targeted tree, as well as those from trees in the adjacent rows. This can sometimes result in the fruits appearing too small in the video frames, leading to inaccurate detection, or causing the same fruit to be counted multiple times as it appears in various frames throughout the mission. Furthermore, the fruit detection process can be compromised by fluctuations in weather conditions, variations in lighting conditions over the course of the day, and the presence of leaves and branches in the camera’s view. To mitigate these challenges and enhance fruit detection accuracy, a refined methodology has been developed specifically for identifying designated structural objects, as represented by the fruits on the trees.

This advanced method requires a digital image of the specified structural objects as its input, expressed as a numerical matrix. Each matrix element corresponds to the brightness level of the associated pixel in the image. In this context, the digital image model for the structural objects of a uniform type is defined through a linear mapping process:

(1) $$f:I\to \langle C_{xy},P_{h\times w}^{i},B_{u\times w}^{i},B_{h\times w}^{i}\rangle ,$$

where $C_{xy}=\left(c_{x},c_{y}\right)$ is the width $\left(c_{x}\right)$ and height $\left(c_{y}\right)$ of the coordinate grid $C_{xy}$ of the coordinate plane *XY* formed to detect structural objects, $P_{h\times w}^{i}=\left(p_{h}^{i},p_{w}^{i}\right)$ is the height $\left(p_{h}^{i}\right)$ and width $\left(p_{w}^{i}\right)$ of the bounding box of the *i*-th target structural object in the *XY* coordinate plane, $B_{u\times v}^{i}=\left(b_{u}^{i},b_{v}^{i}\right)$ are the coordinates of the center of the bounding box of the *i*-th target structural object in the *XY* coordinate plane, and $B_{h\times w}^{i}=\left(b_{h}^{i},b_{w}^{i}\right)$ is the height $\left(b_{h}^{i}\right)$ and width $\left(b_{w}^{i}\right)$ of the bounding box that outlines the *i*-th target structural object in the *XY* coordinate plane.

The coordinates of the center of the bounding box of the *i*-th target structural object according to (1) in the *XY* coordinate plane are calculated using the following formula:

(2) $$\begin{array}{c} b_{u}^{i}=\sigma^{i}\left(t_{x}^{i}+c_{x}\right);\\ b_{v}^{i}=\sigma^{i}\left(t_{y}^{i}+c_{y}\right), \end{array}$$

where $t_{x}^{i}$ and $t_{y}^{i}$ represent the displacement of the center of the bounding box of the *i*-th target structural object from the coordinate grid $C_{xy}$.

The height $\left(b_{h}^{i}\right)$ and width $\left(b_{w}^{i}\right)$ of the bounding box from Formula (2) of the *i*-th target structural object according to (1) in the *XY* coordinate plane are calculated using the following formula:

(3) $$\begin{array}{c} b_{h}^{i}=p_{h}^{i}e^{c_{y}};\\ b_{w}^{i}=p_{w}^{i}e^{c_{x}}. \end{array}$$

**Figure 4:**
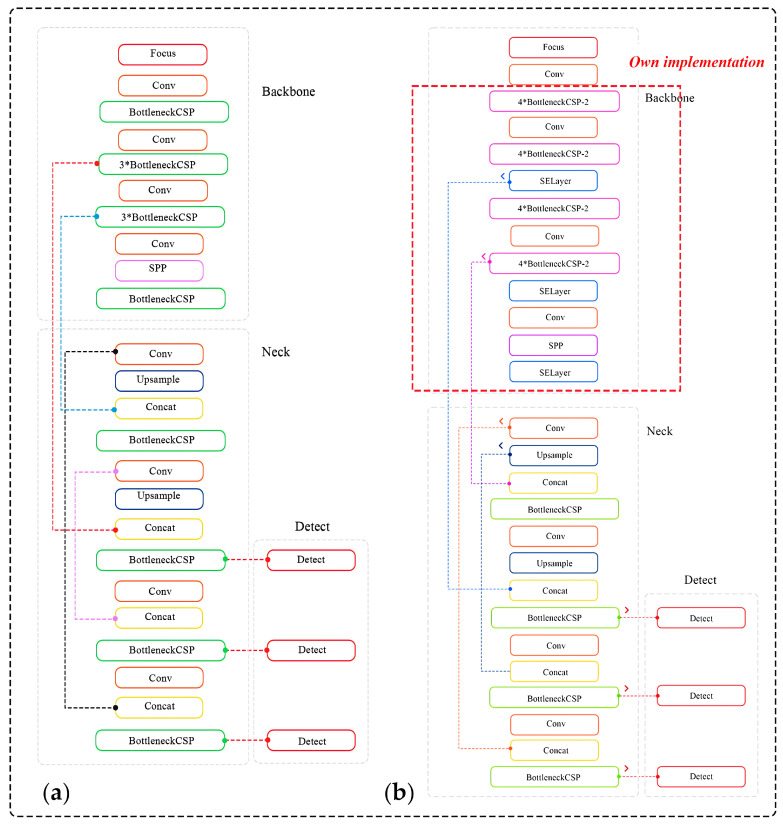
Concise visual comparison of two neural network architectures for fruit detection: (**a**) the original YOLOv5 and (**b**) the proposed YOLOv5-v1. The left panel (**a**) shows the conventional structure of YOLOv5 with repeated bottleneck CSP layers, while the right panel (**b**) illustrates an augmented design of YOLOv5-v1 with additional bottleneck CSP layers and squeeze-and-excitation (SE) layers within the backbone and neck, ending in detection layers. This comparative layout underscores the modifications of the proposed YOLOv5-v1 aimed at improving the feature extraction and inference performance.

The mechanism for overlaying bounding and constraining boxes on the target object is based on the DCNN architecture, which is first used as a baseline and then modified to meet the specified objective. We will henceforth refer to the modified architecture as YOLOv5-v1 (Figure 4).

The hyperparameters for each type of layer involved in the architecture shown in Figure 4 are presented in Appendix C in Table A1.

The initial dimensions of the bounding box applied to the object in the digital image for small and medium scales have been altered. This modification was carried out to enhance the accuracy of detecting each structural object in the image.

Applying the enhanced method under practical conditions results in three main scenarios for placing the designated structural objects within the image: (1) positioned in the foreground of the current task area, (2) situated in the background of the current task area, and (3) located within an additional task area captured in the frame. The original YOLOv5 includes bounding boxes of three different sizes, each assigned to every feature map with preset coefficients.

When fruits are positioned in the background, particularly on trees in more remote rows, the distance to the UAV can be quite substantial, potentially degrading the effectiveness of structural object detection. As a result, such distant specified structural objects in the image should be treated as less reliable targets for both detection and analysis. To reduce the likelihood of mistakenly identifying structural objects in the background of the video frame and to improve the precision of foreground detection, the initial dimensions of the small- and medium-scale bounding boxes were adjusted. This adjustment was based on the specific conditions of the working environment and the outcomes of computational experiments.

The proportions between length and width for both the initial bounding box sizes and the initial sizes of the anchor boxes were established at roughly 1 to 1. Here, the data outline different box types with corresponding values across three feature maps. The values for the bounding boxes are consistently 20, 40, and 80 across feature maps 1, 2, and 3, respectively. Small anchor boxes show a progressive increase in values from feature maps 1 to 3, starting at 80 and 70, then moving to 75 and 75, and finally reaching 85 and 100. Medium anchor boxes display a more varied pattern, with initial values of 95 and 110 in feature map 1, followed by a peak at 130 and 110 in feature map 2, and then slightly reduced values of 115 and 125 in feature map 3. Lastly, large anchor boxes present the highest values, starting with 116 and 90 in feature map 1, escalating to 156 and 198 in feature map 2, and reaching the peak values of 373 and 326 in feature map 3.

Introducing the initial dimensions of both box types is expected to improve the precision of structural object detection in the foreground, while simultaneously diminishing the chances of erroneously identifying irrelevant objects in the background.

**Figure 5:**
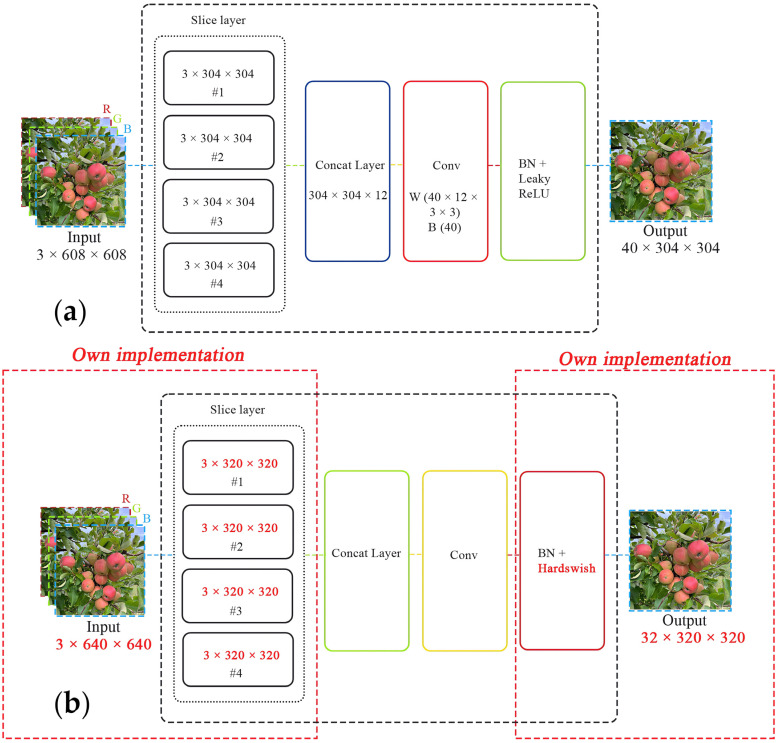
The figure presents two object detection processing pipelines and compares (**a**) a conventional approach of the original YOLOv5 with (**b**) an enhanced implementation of the proposed YOLOv5-v1. Both start with an input image sliced into multiple segments, proceed through the convolution and concatenation layers, and conclude with output images that have undergone batch normalization and activation functions. At the same time, the modified version introduces new dimension values for the input RGB channel (3 × 640 × 640) and feature maps (3 × 320 × 320) in the slice layer and suggests the Hardswish activation function in the focus module, hinting at efficiency gains.

**Figure 6:**
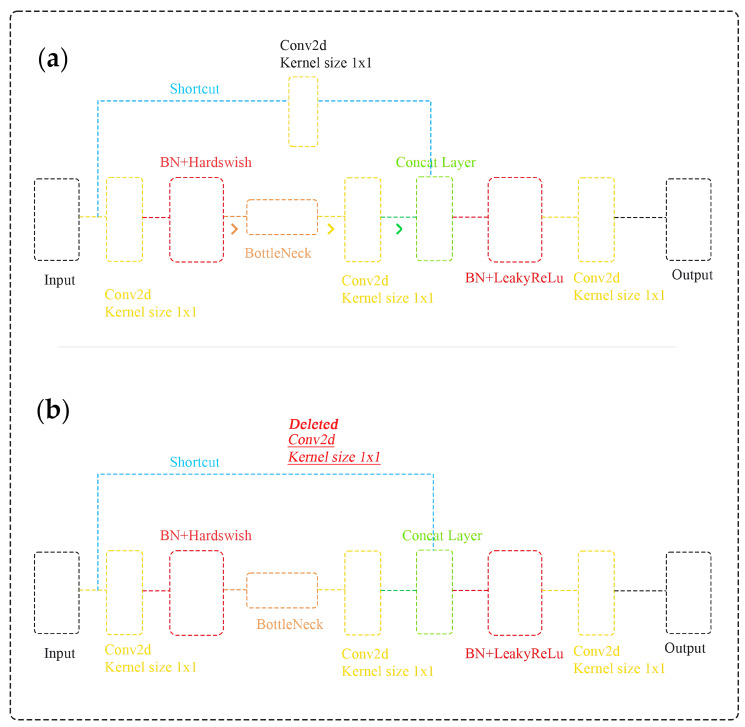
Comparison of two configurations within the BottleneckCSP module: (**a**) YOLOv5 with a shortcut connection and multiple convolutional layers with batch normalization (BN) and Hardswish activation functions, leading to a concatenated layer and output; (**b**) YOLOv5-v1 with one convolutional layer removed, leading to fewer parameters in the module.

To attain remarkable accuracy in real-time structural object detection, it is crucial to significantly reduce the physical size of the original YOLOv5 and fully compress the architecture. Consequently, the primary block of the network, which is responsible for extracting the target object’s features from the input images, underwent modifications to decrease the number of its weight parameters. The enhancements made to the central block incorporate three significant alterations:

1. The focus module of the original YOLOv5 is revamped to expedite the training process, as illustrated in Figure 5. Specifically, the incoming image, presented across three RGB channels with dimensions of 3 × 640 × 640, is segmented into four equal sections, each measuring 3 × 320 × 320. These quartered image segments are then amalgamated into a singular feature map for each RGB channel, culminating in an output dimension of 12 × 320 × 320. Furthermore, a convolutional operation employing 32 kernels is conducted on the newly formed 12 × 320 × 320 feature map, resulting in a feature map sized 32 × 320 × 320. Finally, batch normalization is subsequently applied to this 32 × 320 × 320 feature map, with the normalized output of the same size being transferred to the next processing phase of the network.
2. A bottleneck layer is employed in the cross stage partial (CSP) network. The BottleneckCSP module incorporated into the YOLOv5-v1 architecture, as depicted in Figure 5, entails eliminating the convolutional layer that was previously used in the original BottleneckCSP module to combine the input feature map through a concatenation operation (refer to Figure 6). In the revised BottleneckCSP module, the input feature map is now directly connected to the output feature map depth-wise, leading to fewer parameters in the module.
3. The feature maps are merged. The proposed YOLOv5-v1 employs a technique to merge variously sized feature maps into a single, more discriminative feature map to improve the detection precision of structural objects in video frames. This improvement considers both the simplified design of the updated BottleneckCSP module and the integration of feature maps from the convolutional layers in YOLOv5-v1. Specifically, the original layer mergers from layers 4 to 15, 6 to 11, and 10 to 21 in the original YOLOv5 architecture have been altered to layers 5 to 18, 8 to 14, and 13 to 24 in the enhanced YOLOv5-v1 architecture.

The analysis of images from fruit trees revealed that the structural objects, representing the fruits, predominantly fall into the medium-sized category. Furthermore, the 23rd layer’s output feature map of YOLOv5-v1 is utilized as the input for the classification function targeting these medium-sized objects. To expedite the target object detection process using YOLOv5-v1, the output feature maps from the 14th and 21st layers of the modified architecture were combined.

Because of these adjustments, a more efficient method has been developed. This method automates the conversion of input data, which are provided as digital images of structural objects (fruits on a tree), into output data represented by a set of coordinates that pinpoint the objects’ locations in the image.

#### 2.3.2. Tracking and Counting the Specified Structural Objects

**Figure 7:**
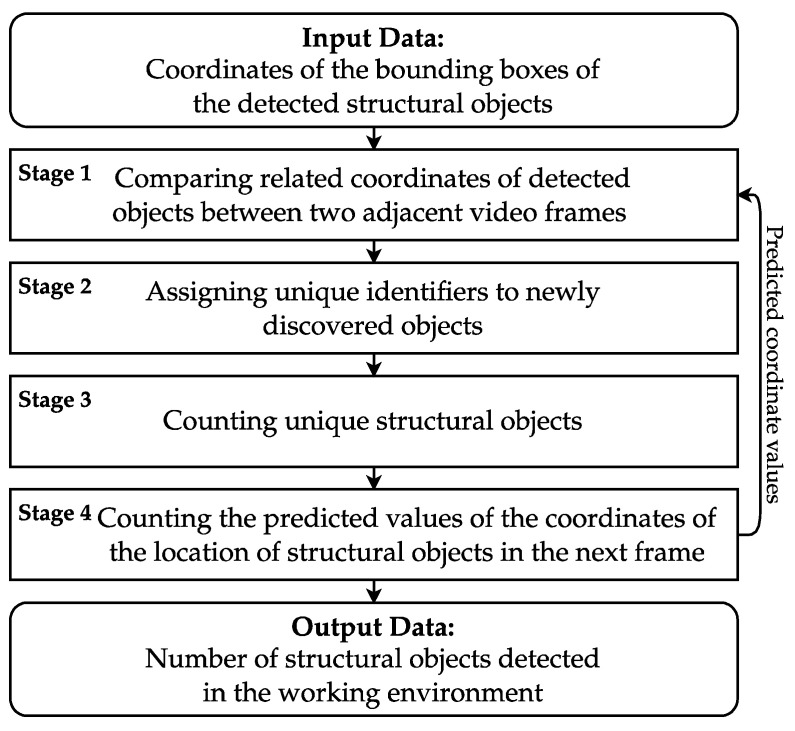
The workflow for tracking the specified structural objects in video analysis. First, the system identifies the coordinates of bounding boxes around the detected objects. These coordinates are compared across successive frames to establish the continuity of the objects. New objects are assigned unique identifiers. The system then counts the distinct structural objects and predicts their subsequent frame coordinates. Finally, the output data enumerate the detected objects within the operational environment.

To accurately count the number of fruits in a video stream, it is crucial to uniquely identify each fruit across two consecutive video frames captured by a UAV. Tracking the fruits is a challenging task due to potential changes in their position and appearance across consecutive frames. These changes could be caused by variations in lighting conditions, camera movements, adjustments to the UAV’s flight path, or other unforeseen environmental influences. The method we have developed for counting these structural objects hinges on correlating the visual characteristics of the detected fruits across consecutive video frames. This correlation considers the unique features of each fruit, their positional changes across frames due to UAV movement, and various environmental factors. A visual representation of this counting method is provided in Figure 7.

The suggested approach unfolds through a series of steps, namely: (1) pre-processing the amalgamated video frames, with bounding boxes highlighting the identified structural objects, (2) systematically comparing the related coordinates of detected objects across two consecutive frames, (3) assigning unique IDs to objects that have just appeared, (4) tallying the unique structural objects, (5) forecasting the positional coordinate values of the objects for the upcoming frame, and (6) generating the final output data, which quantifies the number of structural objects present in the operational environment.

In the Input Data stage (see Figure 7), initial data processing takes place, utilizing updated algorithms from the detection method. This process includes segregating the structural objects into small, medium, and large categories. Small objects are deemed irrelevant for tracking and are consequently excluded from the counting process. The main activity in this phase is the filtration of data, which involves discarding the bounding boxes around the detected small structural objects, resulting in a refined data structure ready for the next phase.

Stage 1 involves the preliminary correlation of the associated coordinates of the detected objects between two successive video frames. Utilizing the data refined in the previous stage, a state is constructed for the detected structural objects in the current video frame. Each object is characterized as follows:

(4) $$\left(x,y,\gamma ,h,v_{x},v_{y},v_{\gamma },v_{h}\right),$$

where (x,y) are the coordinates of the center of the detected object, *γ* is the ratio of width to height of the bounding box of the detected object, and $v_{x}$, $v_{y}$, $v_{\gamma }$, and $v_{h}$ represent the rate of change of the tracking box between consecutive video frames.

Given that numerous objects may be present in a single video frame, it makes sense to establish a probability group predicting the potential location of each identified object in the next frame. By defining the characteristic groups, which are expressed as detected and tracked structural objects using Formula (4), we can create a structured space. In this space, a new subset group is consistently formed, based on algebraic principles. This subset group represents the anticipated location of each detected object, as determined from the previous frame, and is constructed using the Kalman filter [39]. The filter considers the motion state of each object from the previous frame to provide insights into the movement model of the structural object in the current video frame, leading to an optimal estimation of the motion state across the sequence of frames. To facilitate this, the Hungarian algorithm [40] is utilized to calculate the distance matrices between the related coordinates of the detected object locations and the predicted locations from the previous video frame. This calculation considers the variations in input variables and their mutual correlation.

Let $r_{j}$ denote the matrix of characteristic features of each detected structural object in the video frame, and $R_{j}$ the state of all numerical characteristics of detected objects from the preceding 100 frames. Consequently, the minimum distance between the *i*-th feature of the predicted object from the previous video frame and the *j*-th feature of the detected structural object in the current video frame is calculated as follows:

(5) $$d_{\left(i,j\right)}^{\left(1\right)}=\min\left\{1-r_{j}^{T}r_{i}^{\left(k\right)}|r_{i}^{\left(k\right)}\in R_{i},k\in \left(1,100\right)\right\}.$$

The association of two structural objects by distance, using Formula (5), is determined by the intersection of characteristic features, which is defined by a formula with the control threshold $t^{\left(1\right)}$:

(6) $$b_{\left(i,j\right)}^{\left(1\right)}=\{\begin{array}{c} 1,\;d_{\left(i,j\right)}^{\left(1\right)}\leq t^{\left(1\right)};\\ 0,\;d_{\left(i,j\right)}^{\left(1\right)}>t^{\left(1\right)}. \end{array}$$

The result of Formula (6) has a variance value of 1 if the characteristic features intersect, and 0 if there is no such intersection.

The values and motion characteristics of the tracked object between consecutive video frames in a multidimensional space are expressed through the Mahalanobis distance [41], squared between the predicted and detected location of the structural object in the current frame:

(7) $$d_{\left(i,j\right)}^{\left(1\right)}={\left(d_{j}-y_{i}\right)}^{T}S_{i}^{-1}\left(d_{j}-y_{i}\right),$$

where $y_{i}$ is the predicted bounding box values of the object on the current video frame, $d_{j}$ represents the bounding box of the target object on the current video frame, and $S_{i}$ is the covariance matrix for the object on the current video frame.

The process of association by distance (7) with a set control threshold is given by the following formula:

(8) $$b_{\left(i,j\right)}^{\left(2\right)}=\{\begin{array}{c} 1,\;d_{\left(i,j\right)}^{\left(2\right)}\leq t^{\left(2\right)};\\ 0,\;d_{\left(i,j\right)}^{\left(2\right)}>t^{\left(2\right)}. \end{array}$$

After obtaining the movement characteristic values of the tracked object between consecutive video frames using Formulas (7) and (8), we form a numerical matrix based on the Mahalanobis distance, which will determine whether two indicators intersect within the selected region of interest:

(9) $$\begin{array}{c} c_{i,j}=\lambda d_{\left(i,j\right)}^{\left(1\right)}+\left(1-\lambda \right)d_{\left(i,j\right)}^{\left(2\right)};\\ b_{i,j}=\prod_{m=1}^{2}b_{\left(i,j\right)}^{\left(m\right)}. \end{array}$$

The numerical matrix (9) is the result of Stage 1.

Real-time tracking follows a cascading approach, which involves multiple checks for correspondence between the locations of predicted and detected structural objects. Tracking the set of detected objects on a video frame is performed using the intersection over union (IOU) metric. Tracking continues when the IOU value for each detected object achieves the highest numerical value of 0≤IOU≤1.

**Figure 8:**
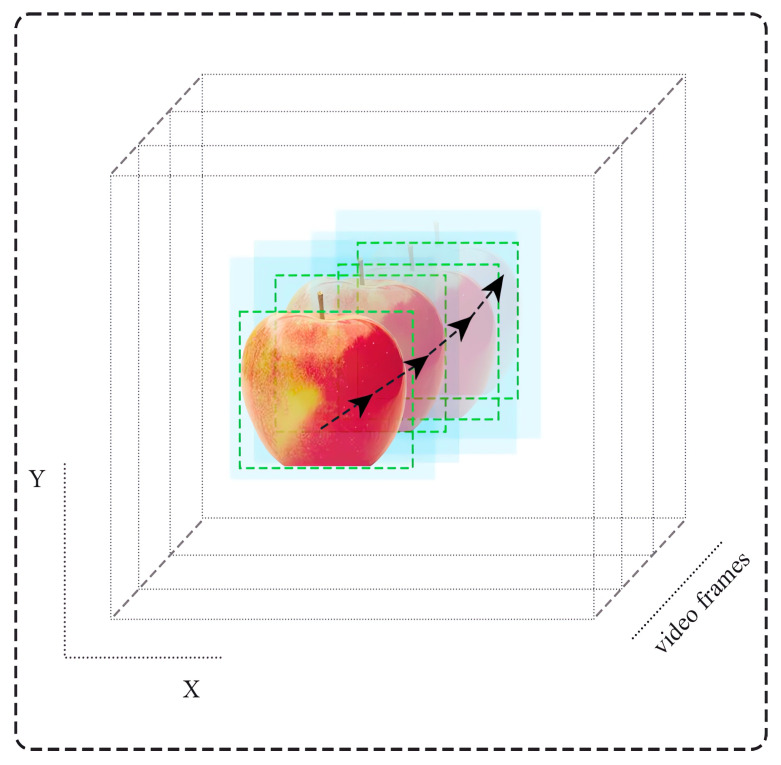
Scheme of the tracking of a structural object across multiple video frames within a three-dimensional coordinate system. Successive frames capture the object’s movement, with each frame layer representing a snapshot in time, and superimposed green bounding boxes tracking the object’s path through space.

The real-time tracking process is illustrated in Figure 8.

After the current phase, a state matrix is generated under Formula (9), which keeps track of objects before passing this information on to the subsequent stages.

In Stage 2 (Figure 7), every newly detected object is assigned a unique identifier. At this stage, a distinctive state characteristic is attributed to each object, supplementing the positional information obtained from Stage 1.

Stage 3 is tasked with counting the unique structural objects. This is accomplished using an object registry, which temporarily stores data about the tracked object, detailing its type, movement, unique identifier, operational status, and time of registration. An object remains “active” as long as it is under the tracking system’s watch. However, if the object disappears from subsequent video frames, the system triggers an event, changing its status in the registry to “inactive.” An “inactive” status indicates that the object has been counted, although it is not then removed from the registry.

**Figure 9:**
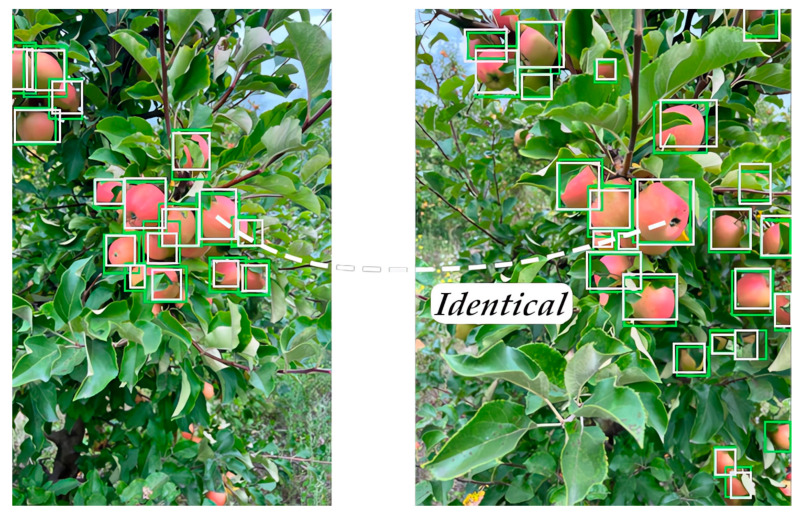
Scheme of YOLOv5-v1’s object detection capability on a fruit tree, with bounding boxes indicating recognized fruits. The left side shows a distorted image, reflecting the data processing stages or errors, while the right side displays clear detection. Green boxes show the algorithm’s accurate fruit detection, while white boxes represent areas the model reconsidered and ultimately deemed below the confidence threshold for fruit classification.

Finally, Stage 4 (Figure 7) aims to predict the future locations of the objects in upcoming video frames. In this stage, we predict where structural objects might be situated across a series of future video frames. If an object stays visible for 100 consecutive video frames, it is considered to be both consistent and unique throughout that period. Figure 9 provides a visual example of how identical structural objects are distinguished.

Moreover, the system initiates an event that leads to the recording of the target object in a temporary registry for objects under tracking. At this juncture, the object is recognized as having been successfully identified and is subsequently added to the aggregated data of the UAV group’s mission. To compute the predictive values, a linear version of the Kalman filter that functions in discrete time intervals is utilized. Through these procedures, a method for counting structural objects is established.

### 2.4. Methodology and Experimental Setting

A general description of the process for conducting computational experiments is presented below.

#### 2.4.1. Experimental Area

The duration of the UAV group’s mission is constrained by the battery’s life and the prevailing weather conditions. The experiments took place at the “SAM+” Farming Enterprise’s apple orchard, located in the village of Kuzmin, within the Horodotska urban territorial community of the Horodotsky district in the Khmelnytskyi region. The orchard primarily features slender spindle apple trees, covering a total area of 50 hectares. The orchard rows vary in length from 500 to 800 m, with trees planted at 2-meter intervals, a 4-meter width between rows, an average tree height of 3.5 m, and a rootstock of 106 mm. The farm estimates that each apple tree produces around 200 apples per season.

**Figure 10:**
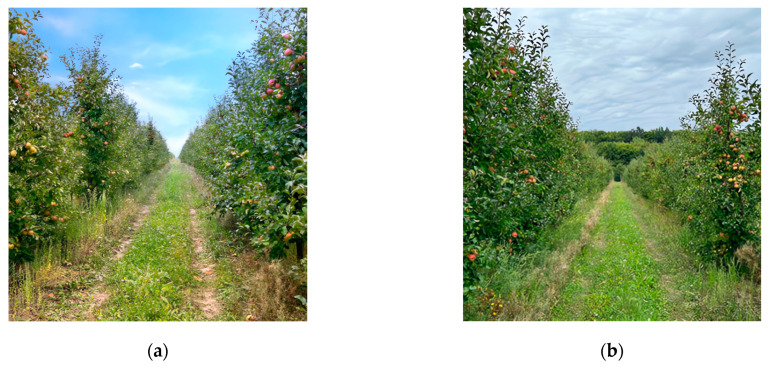
Experimental working environment, photographed under various lighting conditions: (**a**) sunny and (**b**) cloudy.

Experimental investigations within this designated area were conducted under mild weather conditions, including light winds and occasional rain, and during both sunny and cloudy periods. A description of the experimental scenarios is provided below:

(1) On 26 September 2023, between 11:00 and 12:00, an experiment took place in the orchard under clear skies, with the sun positioned directly overhead. Figure 10a showcases a visual representation of the working environment under these sunny conditions.

A general description of the process of conducting experiments for the automated system is described below:

(2) On 28 September 2023, from 13:00 to 14:00, an experiment took place in a fruit orchard under cloudy conditions, with the sun obscured by clouds. Figure 10b illustrates a sample of the working environment during such overcast conditions.

(3) Another experiment was executed on 29 September 2023, from 19:00 to 20:00, in sunny weather with the evening sun near the horizon. The setting sun resulted in a significant shading effect on the fruits within the apple orchard.

We configured the UAVs and their cameras by setting the following parameters: the flight height, the observation angle of the UAV-based camera, and the ISO (International Organization for Standardization) [42] settings of the camera (Table 1).

The effectiveness of the experimental study hinges on whether the working area has been previously utilized for experiments. In this regard, two rows of fruit trees were selected from the working environment, comprising approximately 1600 m of the UAV’s flight path and encompassing an estimated 800 trees. This route has been traversed multiple times for the experiments.

#### 2.4.2. Equipment Details

Setting up the necessary equipment in the working environment is crucial. This step involves defining the technical specifications of each hardware device, which will influence the future efficiency of the working mission’s execution. The selected equipment and its technical specifications are summarized in Table 2.

Table 2 comprises the technical specifications of the selected equipment and their relevance to conducting precise and efficient missions in apple orchards, primarily focusing on fruit detection using UAVs equipped with advanced RTK modules and robust power supplies. The integration of these elements is crucial for real-time fruit harvest monitoring and when leveraging multiple UAVs for the acquisition of high-quality images.

#### 2.4.3. Evaluation Criteria

In this study, we assessed model performance using several metrics [43].

Precision indicates that the majority of the fruits identified by the system are indeed correct. Precision is calculated using the following formula:

(10) $$\mathrm{Precision}=\frac{\mathrm{TP}}{\mathrm{TP}+\mathrm{FP}},$$

where TP (true positive) refers to correctly predicted positive cases, and FP (false positive) refers to negative cases that are wrongly classified as positive.

Recall complements precision by quantifying the system’s ability to identify all relevant instances of fruit within a dataset. Recall is calculated as:

(11) $$\mathrm{Recall}=\frac{\mathrm{TP}}{\mathrm{TP}+\mathrm{FN}},$$

where FN (false negative) represents those positive cases incorrectly labeled as negative.

The F_1_-score is a balance between precision and recall, with a range from 0 (worst) to 1 (best). It is computed using the following formula:

(12) $$F_{1}\text{-}\mathrm{score}=2\times \frac{\mathrm{Precision}\times \mathrm{Recall}}{\mathrm{Precision}+\mathrm{Recall}}.$$

In this study, we focused on a single category, “Fuji apples”, for which the mean average precision (mAP) is calculated as follows:

(13) $$\mathrm{mAP}=\frac{1}{n}\sum_{i=1}^{n}{\mathrm{AP}}_{i},$$

where ${\mathrm{AP}}_{i}$ is the average precision for the *i*-th threshold, representing the area under the precision-recall curve. This is further defined as:

$$\mathrm{AP}=\int_{0}^{1}\left(\mathrm{Precision}\times \mathrm{Recall}\right)\;dx.$$

The false positive rate (FPR) is defined as the ratio of the number of non-defective fruits that are wrongly categorized to the total number of actual non-defective fruits. FPR is formalized as:

(14) $$\mathrm{FPR}=\frac{\mathrm{FP}}{\mathrm{FP}+\mathrm{TN}}.$$

where FP represents the number of false positive cases and TN stands for the number of true negatives.

The false negative rate (FNR) measures the proportion of defective fruits that are mistakenly labeled as non-defective. FNR is formalized as:

(15) $$\mathrm{FNR}=\frac{\mathrm{FN}}{\mathrm{FN}+\mathrm{TP}}.$$

FPR and FNR both represent the error rates made by the evaluated detectors.

We utilized the average detection speed, measured in seconds per picture, to assess the system’s efficiency. Moreover, the number of model parameters and the model’s size, measured in megabytes, were used to evaluate the system’s complexity and resource requirements. A system with fewer parameters and smaller size is generally more desirable, as it indicates a leaner, more efficient model that can be deployed more easily in various hardware setups, a key consideration in Industry 4.0 environments.

Overall, the utilized criteria offer a comprehensive framework for evaluating fruit detection systems in the context of intelligent sensors and advanced computing. They address both the precision and efficiency aspects, which are critical in industrial applications where speed, precision, and resource optimization are essential.

## 3. Case Study

### 3.1. Creating a Detector for Tracking Tasks

The success of detecting and counting structural objects in the experimental environment is partly contingent on the preliminary stage of preparing the training data.

#### 3.1.1. Training Dataset

Fuji apples, characterized by their striped raspberry-colored blush, yellow subcutaneous spots, and rounded shape, were used for training YOLOv5-v1. The images were captured under varying weather conditions and distances using an iPhone 13 Pro Max [44] smartphone camera.

Considering the working environment’s challenges, such as organic objects being obscured by foliage and varying natural conditions, 1214 images of apples were taken under specific conditions to enhance the system’s applicability. These conditions included various forms of occlusion and lighting angles.

The dataset was divided into 200 images for testing (equally split between sunny and cloudy conditions) and 1014 images for training. A detailed distribution of this fruit image dataset is shown in Table 3.

The images captured were subjected to augmentation and enhancement through CLAID.AI technology (San Francisco, CA, USA) [45], which involved operations such as increasing and decreasing the brightness, horizontal and vertical flipping, and rotation at various angles. Additionally, images featuring apple fruits of diverse colors and shapes were incorporated. Consequently, the final dataset comprised 16,224 images of fruits, serving as the definitive set of training data. This set included 15,210 enhanced images and 1014 raw images, ensuring that there was no overlap between the training and test datasets.

After the data preparation and processing, the task was to append one or more descriptive and meaningful labels to the images, adding context and facilitating more effective model training. For this, we utilized the Amazon SageMaker Data Labeling tool (Seattle, WA, USA) [46] to prepare a dataset with superimposed labels. Out of this dataset, 200 test images were manually labeled, while the remaining images were annotated automatically.

It is noteworthy to emphasize that Amazon SageMaker’s accuracy primarily influences the quality of prepared data. However, this influence does not inherently compromise the precision of the object detection model itself. The accuracy of the model depends on numerous factors, including the diversity and representativeness of the training data, the robustness of the model architecture, and the effectiveness of the training process. In this case, the rigorous data augmentation and the diversified conditions under which the images were captured aim to enhance the model’s generalizability and robustness. Therefore, while annotation accuracy is crucial to ensure that the model learns from correct examples, it is the comprehensive training process, the diversity of the dataset, and the robustness of the model architecture that collectively determine the accuracy of the object detection outcomes.

During the initial training stage, the Adam optimizer was employed over 30 epochs with a set learning rate of 0.001. The total duration of training was approximately 9 h. The hardware setup for the training and testing included an Intel (R) Core (TM) I7-9750H processor running at 2.6 GHz, 32 GB of RAM, and an NVIDIA GeForce RTX 2060 graphics processor with 6 GB of video memory. The software environment included programming language Python v3.10.13 (Wilmington, DE, USA), PyTorch v1.13.1 (New York City, NY, USA) [47], CUDA v11.7 (Santa Clara, CA, USA) [48], and OpenCV v4.7.0 (free and open-source software under Apache 2 License) [49] toolkits, all operating on the Windows 11 platform (Redmond, WA, USA).

#### 3.1.2. Creating a Detector

**Figure 11:**
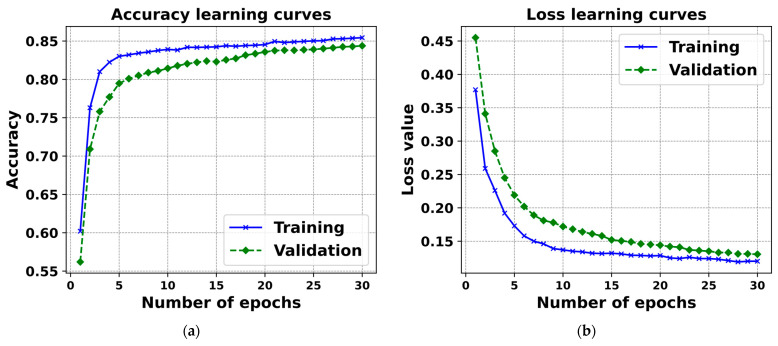
This figure presents the learning curves for (**a**) accuracy and (**b**) loss in YOLOv5-v1’s training and validation phases over 30 epochs. The accuracy curve (**a**) demonstrates rapid improvement and subsequent stabilization, indicative of effective learning, while the loss curve (**b**) shows a steep decline before plateauing, suggesting a reduction in model error.

For assessing the constructed detector, we selected evaluation metrics such as mean precision, recall, F_1_-score, and mAP, formalized by Formulas (10)–(13), respectively. The outcomes of the training, leveraging YOLOv5-v1, are illustrated in Figure 11.

The values of the loss function for both the validation and training datasets, as depicted in the curves in Figure 11, show a rapid decline during the initial 100 iterations of the YOLOv5-v1’s training. A steadier pattern starts to form after 250 iterations, leading to the establishment of the model after 300 training iterations. The trends indicate that YOLOv5-v1 has been adequately trained without overfitting (see Figure 11).

**Figure 12:**
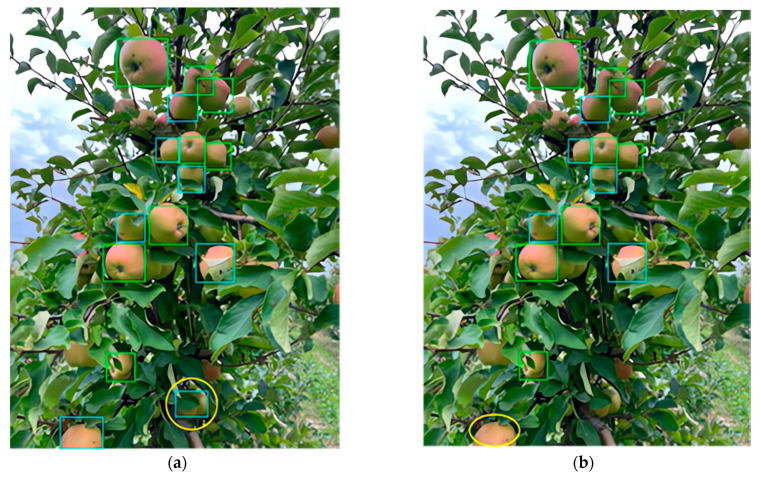
An output of YOLOv5-v1 examining a fruit tree, representing true positives, i.e., successful fruit identification, with green bounding boxes and false positives, i.e., incorrectly identified an object as a fruit when it is not, with blue boxes. In panel (**a**), one apple at the bottom is circled in yellow, suggesting an omission in detection. In contrast, panel (**b**) shows the same scene without the omission, indicating a refined detection process.

Upon identifying a specific group of structural objects within a video frame, YOLOv5-v1 applies a filtering process based on a predetermined prediction confidence threshold. The precision and completeness of the detection outcomes vary, depending on the single recognition model and the different confidence threshold values that are set. Inappropriate settings of the model’s confidence threshold can lead to unpredictable outcomes (see Figure 12).

If the confidence threshold is set exceedingly low, objects in the foreground that are erroneously detected are highlighted (and marked with a yellow ellipse in Figure 12a). Conversely, if the probability threshold is excessively high, the target structural object in the foreground might be missed or incorrectly detected, as indicated by the yellow ellipse in Figure 12b.

**Figure 13:**
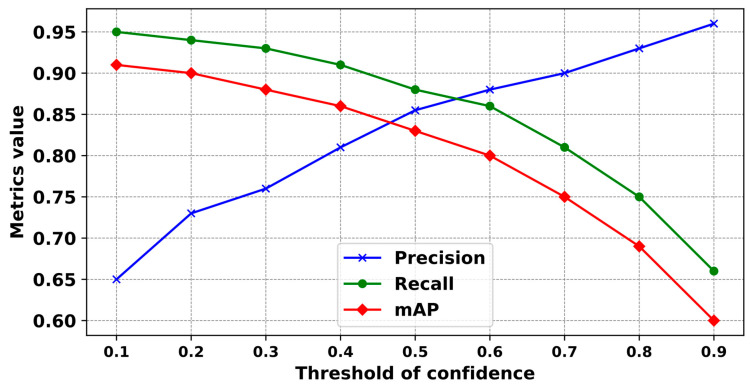
The validation curves of precision, recall, and mAP of YOLOv5-v1 against varying confidence thresholds. Precision (blue) rises with the threshold, indicating fewer false positives at higher confidence levels. Recall (green) decreases, suggesting that more true positives are missed as the threshold increases. The mAP (red) curve peaks at a mid-range threshold, balancing precision and recall. This graph suggests an optimal threshold of 0.5 for the balance between detecting as many structural objects as possible while maintaining high accuracy.

The results from changing the efficiency of YOLOv5-v1 with different confidence thresholds are illustrated in Figure 13.

The confidence threshold was determined based on the following scenarios:

(1) When the confidence threshold was established at below 0.5, YOLOv5-v1 showed suboptimal recognition precision, falling below 80%.
(2) Conversely, setting the confidence threshold above 0.5 led to a gradual decrease in the average precision of the classification.
(3) Optimal efficiency and performance from YOLOv5-v1 were achieved when the probability threshold was precisely set at 0.5. Under this setting, YOLOv5-v1 attained average classification accuracies of 83.8%, 91.5%, and 86.8% for three object categories that correspond to three types of anchor boxes: small, medium, and large (see Section 2.3.1).

### 3.2. Test Results of the Created Detector

The efficacy and performance of the proposed YOLOv5-v1 were extensively evaluated, based on its ability to detect objects across a set of 200 test images. Within these images, there were 2336 instances of fruits; 1007 of these were deemed capturable, whereas 1329 were classified as non-capturable. Detailed results of the recognition performance achieved by the proposed method are shown in Table 4.

The data (see Table 4) reveal the performance metrics of the proposed YOLOv5-v1 model in recognizing fruits. Specifically, for the fruits deemed capturable, YOLOv5-v1 demonstrated a precision value of 85.5%, a recall value of 94.3%, a mAP value of 89.2%, and an F_1_-score of 89.7%. Conversely, the performance of YOLOv5-v1 on ambiguous fruits yielded precision of 82.6%, recall of 89.3%, a mAP value of 84.9%, and an F_1_-score of 85.8%. When considering object detection, YOLOv5-v1 achieved overall performance metrics of 83.8% precision, 91.5% recall, 86.8% mAP, and an F_1_-score of 87.5%.

**Figure 14:**
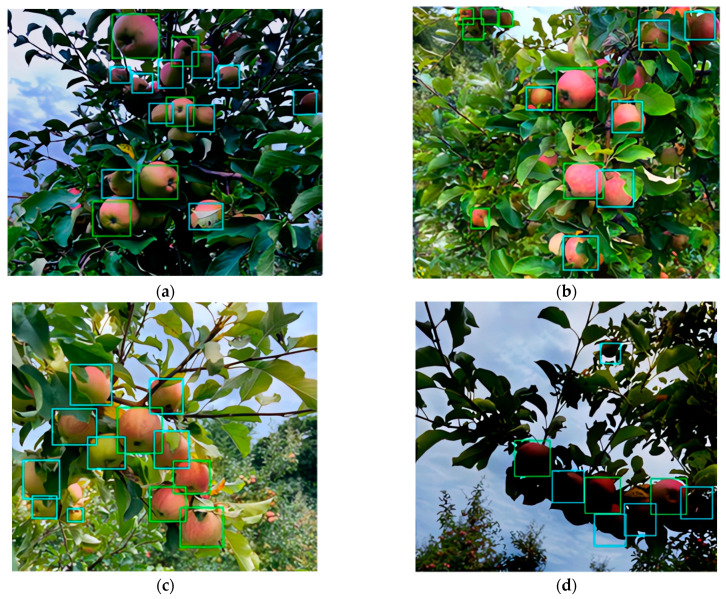
This figure demonstrates the visual performance of YOLOv5-v1 in fruit detection under varied lighting conditions. Figure (**a**) exhibits its robust detection amidst cloud cover, (**b**) shows its accuracy with side light, (**c**) reveals the impact of backlighting on detection performance, and (**d**) indicates the challenges and potential overexposure when in direct sunlight. Green and blue boxes in these figures represent true positive and false positive cases, respectively.

Figure 14 showcases the ability of YOLOv5-v1 to differentiate between fruits that are capturable and those that are not, all under a variety of weather and lighting conditions.

In the visual labels, green bounding boxes represent objects that could be captured, whereas blue bounding boxes denote objects that were not captured. As illustrated in Figure 14c,d, the proposed YOLOv5-v1 can identify structural objects in images, even in varied lighting conditions such as uniform lighting, overcast skies, and direct sunlight. Additionally, YOLOv5-v1 demonstrates proficiency in detecting structural objects under different lighting orientations, including front, back, and side lighting in sunny conditions.

Based on the assessment data from the “SAM+” Farm, the orchard’s fruit count at the time of the experiment was estimated to be around 160,000. The findings from the fruit yield detection and quantification are detailed in Table 5.

The conducted experiments yielded results wherein 147,382, 145,223, and 132,304 pieces of fruit were successfully identified and tallied across three distinct weather conditions (see Table 5). Simultaneously, it was noted that there were instances of fruit detection without counting, with numbers amounting to 72,143, 69,944, and 69,336 for each weather condition, respectively. Despite being recognized as fruits by YOLOv5-v1, these items were not counted due to their location outside the designated operational zones.

As can be seen from Table 5, the specific quantities of fruit, numbering 5743, 6611, and 8442 corresponding to the three different weather scenarios, were concurrently detected by cameras from two or three UAVs but were only counted once. This counting approach aligns with the logic of the proposed method for tallying structural objects. However, it is worth noting that some fruits were either obscured by foliage or situated in areas that were challenging to perceive, both for the UAV camera and by human observation. Consequently, the system’s detector made a minor number of errors.

Table 6 displays the statistical metrics used to evaluate the performance of fruit detection.

**Figure 15:**
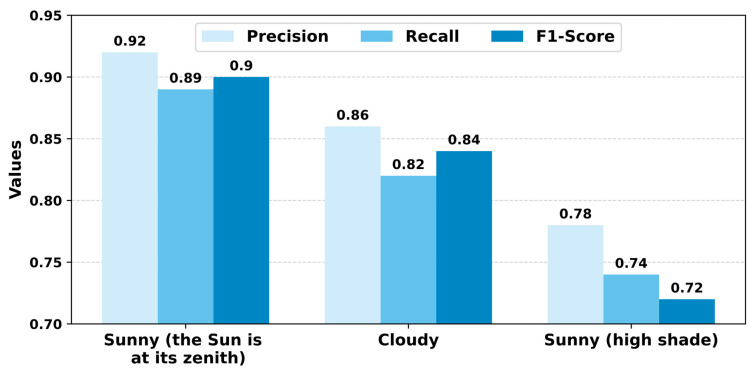
A comparison by evaluation criteria of the performance of YOLOv5-v1 under different lighting conditions. Precision, recall, and F_1_-score metrics are compared across sunny, cloudy, and shaded environments. High precision during sunny conditions suggests fewer false positives, while lower scores in shaded conditions indicate increased difficulty in object identification. The F_1_-score, a harmonic mean of precision and recall, reflects overall accuracy, peaking under sunny conditions.

As can be seen from Table 6, the peak metric values were attained when the weather was sunny and the sun was directly overhead, while the lowest values occurred under heavy shading conditions. Figure 15 illustrates a visual comparison of the recognition criteria under various weather conditions.

The experimental results demonstrate that YOLOv5-v1 achieves precision levels of (1) 92.1% in sunny conditions, (2) 90.8% on cloudy days, and (3) 82.7% in conditions that are sunny but with substantial shading. These outcomes underscore the impressive performance of our approach in real-world scenarios for fruit detection and counting.

**Figure 16:**
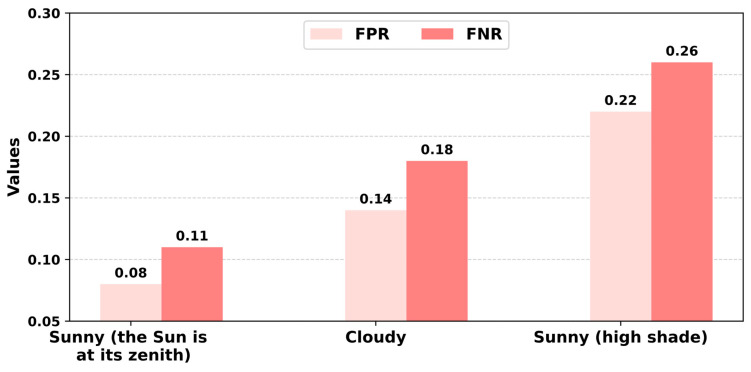
A comparative analysis of the false positive rate (FPR) and false negative rate (FNR) for YOLOv5-1 under various lighting conditions. The model achieves the lowest FPR under direct sunlight and the highest FNR in deep shade, implying a tendency to miss detections rather than make erroneous ones in difficult lighting.

A comparative analysis of the error rates associated with the UAV group across various meteorological conditions is illustrated in Figure 16.

As can be seen from Figure 16, the error rate of fruit recognition in real-world scenarios is highly influenced by weather conditions. This may be due to certain work areas being obscured by shadows from nearby trees. This issue, along with visual interference from various objects like leaves and branches, can considerably hinder the system’s ability to identify and track target objects in real time, particularly given the restricted field of view of UAV cameras.

The proposed YOLOv5-v1 delivers high recognition precision and embodies the attributes of a lightweight CNN, standing out as the most efficient among the five compared models in terms of the highest mAP value. This advantage is particularly beneficial for real-time operations involving multiple UAVs.

To assess the efficiency of the real-time video stream synchronization method developed for this study, the structural similarity index measure (SSIM) and peak signal-to-noise ratio (PSNR) were utilized [50]. A high-efficiency synchronization is indicated by an SSIM index ranging from 0.5 to 1, while a value between 0 and 0.49 denotes inefficiency. The PSNR index, on the other hand, gauges the quality of images resulting from the merging process, with higher values indicating superior image quality.

The performance of the video stream synchronization module within the approach, applying the developed method across 12 sets of sequential video frame groups, is shown in Table 7.

The performance metrics from Table 7 indicate that the SSIM index for efficiency fluctuates between 0.45 and 0.91, with an average value of 0.87. Images registering an SSIM index below 0.50 are flagged as distorted by the system. Simultaneously, merged images with a PSNR index above 30 are deemed of high quality, whereas a PSNR value under 30 suggests inferior image quality, potentially due to external environmental influences such as strong winds or rain. An image with an SSIM index under 0.50 and a PSNR index below 30 is categorized as distorted and is subsequently excluded from further processing in object detection and enumeration.

Finally, to evaluate and validate the performance of YOLOv5-v1, it was tested on 200 images from the test set and compared with recent related detection algorithms: (i) Mai et al. [34] employed Faster R-CNN; (ii) Chu et al. [35] adopted Mask R-CNN; (iii) Biffi et al. [36] used ATSS, ResNet50, and FPN; (iv) Sun et al. [37] utilized the modified YOLOv5-CS (Table 8). The evaluation was performed based on the values of mAP and the average recognition speed.

As can be seen from Table 8, the proposed YOLOv5-v1 model outperforms four other approaches. In particular, it achieves the highest mAP rate of 86.8%, indicating superior precision in detecting and counting fruits. Its detection speed is also acceptable at 0.015 s per picture, striking a balance between precision and efficiency. Moreover, it has a low model size of 12.70 MB and a moderate number of parameters (6.52 × 10^6^), making it highly efficient in terms of computational resources.

Consequently, the YOLOv5-v1 model demonstrates a significant advancement in fruit detection technology. It not only offers the highest mAP rate but also maintains a balance between detection speed and resource efficiency. This makes it particularly suitable for Industry 4.0 applications where precision, speed, and resource optimization are key factors. The YOLOv5-v1’s attributes align well with the demands of modern, intelligent sensor systems, marking it as a leading choice in the domain.

In summary, the system’s performance was appraised using several criteria: (i) statistical metrics, including mAP in fruit detection, FPR, and FNR, and (ii) real-time video frame synchronization efficiency indicators, SSIM, and PSNR. The experiments validate the efficiency of YOLOv5-v1, underscored by an 86.8% mAP rate in fruit detection and counting.

## 4. Discussion

This paper contributes to the field of intelligent sensors and advanced computing in the era of Industry 4.0 by expanding upon Reference [51] in the domain of digital agriculture, specifically focusing on the integration of UAVs and new data processing methods. The presented research outlines a novel approach for the dynamic capture of images using multiple UAVs, ensuring synchronization between different UAVs and autonomous movement. This is a considerable enhancement over the findings in [51], which relied on a single camera perspective and did not address the challenges of synchronizing data from multiple sources. Moreover, this study focuses on the development of a system that excels across various fruit varieties and orchard configurations and offers computational efficiency for real-time applications, which is crucial for digital agriculture. This focus on computational efficiency and adaptability to different environmental conditions is a notable improvement over the previous work, which mainly concentrated on enhancing precision in recognizing fruits on orchard trees.

The core scientific novelty of this work lies in the deployment of a multi-UAV system for the dynamic capture and processing of orchard images in the domain of digital agriculture, which also contributes to the advancement of smart sensors and advanced computing in the Industry 4.0 era. This system ensures real-time data synchronization and autonomous movement, addressing the intricacies of managing multiple data sources and navigating orchard environments effectively. Another novelty that this study presents is the integration of advanced data processing methods, notably the YOLOv5-v1 model, which provides high precision in fruit detection and counting. The model’s robust performance under varying environmental conditions and its adaptability to different fruit varieties and orchard configurations demonstrate a prominent development compared to the existing methodologies. The proposed novelties enhance the efficiency and precision of orchard management and fit perfectly with the needs and challenges of modern digital agriculture, paving the way for more reliable and comprehensive agricultural solutions. All these described contributions align well with the core tenets of Industry 4.0, which emphasizes automation, data exchange, and manufacturing technologies.

The empirical results of our system deployment are promising, with a mAP rate of 86.8% in fruit detection and counting, surpassing the existing analogs. False positive and negative rates under various environmental conditions were thoroughly assessed: 8% and 11.5% under sunny conditions, 14.7% and 18.3% in cloudy weather, and 22.2% and 26.2% in shaded areas, respectively. These results demonstrate the system’s ability to identify and count fruits with a considerable degree of precision. The utilization of multiple UAVs allows for extensive orchard coverage, surpassing the limitations of single-UAV or conventional ground-based approaches.

The strengths of the proposed approach lie in its capacity to dynamically capture and process images from numerous UAVs, ensuring real-time data synchronization and processing. This capability is crucial for the timely detection of fruits and is essential for efficient orchard management and harvest planning. Moreover, the integration of image quality optimization techniques mitigates common challenges in orchard environments, like variable lighting and obstructions from foliage. When compared to the state of the art, the proposed approach exhibits significant improvements in various aspects. Traditional manual counting methods are labor-intensive and error-prone, while existing automated systems often struggle with natural orchard complexities, such as uneven lighting and physical obstructions. Furthermore, various DCNN models currently employed in fruit detection face obstacles in terms of computational efficiency and environmental adaptability. In contrast, the proposed approach introduces a novel method for real-time video stream synchronization, one that is specifically designed to be resilient against these challenges.

Despite the highlighted improvements, the authors acknowledge certain limitations. The performance of the YOLOv5-v1 model may be impacted by visual obstacles in orchards, such as the obscuring of fruits by foliage. The system’s reliability under extreme environmental conditions like heavy rain or fog has yet to be thoroughly examined. Moreover, the adaptability of this system across various fruit types and orchard configurations warrants further exploration. Nevertheless, the substantial achievements garnered through carrying out these experiments, including a high mAP rate, low error rates, and a compact model size under diverse conditions, underscore the robustness and potential of the YOLOv5-v1 model in enhancing orchard management.

In comparison to previous studies in the field, the proposed approach introduces noteworthy advantages. The study by Mai et al. [34] presents a Faster R-CNN with classifier fusion for the automatic detection of small fruits. While their model offers a significant contribution to fruit detection technology, the proposed multi-UAV approach provides broader coverage and real-time data processing capabilities, which are crucial for comprehensive orchard management. Chu et al. [35] explore apple detection using a suppression Mask R-CNN. Their model achieves good performance in fruit detection, but the proposed YOLOv5-v1’s strength lies in the synchronized capture and processing of images from multiple UAVs, ensuring higher precision and efficiency. Biffi et al. [36] introduce an innovative ATSS DL-based approach for apple fruit detection. Conversely, our study aligns with the trajectory of employing DL techniques for fruit detection and extends it by integrating real-time video stream synchronization and tackling the challenges of environmental adaptability. Lastly, the study by Sun et al. [37] presents a lightweight algorithm for apple detection based on an improved YOLOv5 model that achieves an impressive detection speed of 0.013 s/pic. However, our approach complements this by offering a system that focuses not only on lightweight model architecture that detects fruits relatively rapidly in real-time processing but also achieves a higher detection accuracy (a mAP rate of 86.8% by YOLOv5-v1, compared to 81.7% by Sun et al.).

In summary, the proposed multi-UAV imaging and DL approach for fruit recognition in orchards marks a significant improvement over the existing solutions. It offers high precision and real-time processing capabilities, aligning with the needs of modern digital agriculture. Nonetheless, addressing the challenges related to environmental conditions and enhancing system reliability will be essential for the broader implementation and success of this technology in the agricultural sector.

## 5. Conclusions

In the innovative landscape of Industry 4.0, this study introduces a novel approach for real-time fruit detection and counting. A key advantage of the proposed approach is its ability to instantly collect and synchronize video footage from several UAV-mounted cameras, merging this footage into a single, coherent data structure that is subsequently transformed into an uninterrupted visual stream. By incorporating image quality improvement functions, our approach ensures the most effective identification of target objects during UAV operational tasks.

The results of the study confirm the high efficiency of the introduced YOLOv5-v1, with a high mAP rate of 86.8% outperforming its analogs in fruit detection and counting. This is in addition to relatively low average error rates of 14.7% for FPR and 18.3% FNR obtained in typical cloudy weather, illustrating the capability of the proposed YOLOv5-v1 to recognize apples within orchard environments effectively. The technique demonstrates strong video stream synchronization, as indicated by SSIM index values between 0.45 and 0.91, averaging at 0.87, and PSNR index values from 27.2 to 39.1, which points to the excellent quality of the combined images. Nonetheless, overcoming the challenges posed by environmental factors and enhancing system reliability remain critical for the broader application and success of this technology in agriculture.

Future research will be focused on improving the model’s resilience in adverse weather conditions, ensuring consistent performance across various environmental settings. Furthermore, the adaptability of the system to different types of fruits and orchard layouts is an appealing area of exploration to enhance its utility in diverse agricultural scenarios. This aligns with the overarching goals of Industry 4.0, where the integration of intelligent sensors and advanced computing systems is paramount in transforming traditional agricultural practices.

## Figures and Tables

**Table 1:** UAV flight parameters for agricultural imaging, detailing the positions, corresponding flight heights, camera observation angles, and ISO settings for varying lighting conditions.

UAV Position	Flight Height, Meters	Observation Angle, Degrees	ISO Setting of the Camera
Bottom	1.2	25 upward	(1) 400 on a sunny day; (2) 800 on a cloudy day; (3) 1600 during late evening
Middle	2.2	0	
Top	3.2	20 downward	

**Table 2:** Technical details of the fruit detection equipment that was used in the working environment.

Category	Equipment	Details	Relevance to Mission
Base Station	D-RTK 2 High Precision GNSS Mobile Station	Portable and lightweight Data transmission: 1 Hz to 10 Hz Frequencies: L1, L2, L5, L-band Positioning accuracy: <2.5 cm horizontal, <5 cm vertical	Enables mobility and precise positioning in various orchard segments
TK Modules	Multi-layer GNSS antennas, Radio modems	Broad signal reception angle Receives GPS signals Facilitates rapid communication among components	Ensures precise 3D GPS coordinates Enables real-time and high-accuracy UAV movement
Power Supply	External batteries for UAVs	Provides up to 1 h of operational time	Sustains prolonged UAV operations in the field
UAV Group	DJI Phantom 4 RTK UAVs	High-resolution camera 30-min autonomous operating time Resilient to adverse weather and wind gusts	Conducts high-precision monitoring and fruit detection Ensures safe and efficient mission execution in varying conditions

**Table 3:** The distribution of a 200-image dataset used for fruit detection testing under varying lighting conditions. It documents the counts of images with clearly visible fruits and those with mixed occlusion, highlighting a higher frequency of occlusion during sunny conditions. The dataset comprises an equal number of images from sunny and cloudy conditions, with a total of 1007 instances of pronounced clearly visible fruits and 1329 instances with mixed occlusion.

Data	Sunny	Cloudy	Total
Number of images	100	100	200
Images with clearly visible fruits	482	525	1007
Images with mixed occlusion	766	563	1329

**Table 4:** The performance results of YOLOv5-v1 for fruit detection. The table presents precision, recall, F_1_-score, and mAP for those structural objects that can and cannot be captured. The data reveal higher precision and recall for detectable objects, with the overall performance showing 83.8% precision, 91.5% recall, and a balanced 87.5% F_1_-score across 2336 objects, indicating both the system’s effectiveness and areas for improvement in fruit detection accuracy.

Data	Number of Objects	Precision (%)	Recall (%)	F_1_-Score (%)	mAP (%)
The amount that can be captured	1007	85.5	94.3	89.7	89.2
The amount that cannot be captured	1329	82.6	89.3	85.8	84.9
Total	2336	83.8	91.5	87.5	86.8

**Table 5:** A comparative analysis of fruit detection and counting under various lighting conditions using multiple UAVs. It quantifies the fruits that are detected and counted, those detected but not counted, and instances where fruits were detected by multiple cameras but were counted only once.

#	Weather Conditions	Detected and Counted (Number)	Detected and Not Counted (Number)	Detected by Many Cameras, but Counted Once (Number)	Total (Number)
1	Sunny (sun at the zenith)	147,382	72,143	5743	~160,000
2	Cloudy	145,223	69,944	6611	~160,000
3	Sunny (strong shade)	132,304	69,336	8442	~160,000

**Table 6:** This table quantifies the precision, recall, F_1_-score, FPR, and FNR of the proposed YOLOv5-v1 under different lighting conditions. It shows that performance peaks with direct sunlight, declines under cloud cover, and is lowest in strong shade.

#	Weather Conditions	Precision (%)	Recall (%)	F_1_-Score (%)	FPR (%)	FNR (%)
1	Sun (sun at the zenith)	92.1	89.3	90.5	8	11.5
2	Cloudy	86.1	82.1	84	14.7	18.3
3	Sun (strong shade)	78.1	74.2	72.4	22.2	26.2

**Table 7:** Research results on the effectiveness of the video stream synchronization method, as obtained by a group of UAVs. Each group, randomly selected for testing, comprises three video frames captured by three different UAVs, which are then merged into a single image. The table presents an evaluation of twelve randomly combined images, using SSIM and PSNR as quality metrics.

The Index of Combined Image (#)	SSIM	PSNR	The Index of Combined Image (#)	SSIM	PSNR
01	0.90	35.2	07	0.86	31.4
02	0.45	27.2	08	0.47	27.9
03	0.72	37.5	09	0.90	30.1
04	0.85	29.1	10	0.83	31.5
05	0.87	36.9	11	0.52	28.9
06	0.91	39.1	12	0.86	30.4

**Table 8:** A comparison of various object detection approaches. Metrics compared include mAP, average detection speed, the number of parameters, and model size. The bolded numbers indicate the best-performing approach.

Object Detection Models	mAP (%)	Average Detection Speed (s/pic)	Number of Parameters	Model Size (MB)
Mai et al. [34]	71.8	0.053	6.15 × 10^7^	235.00
Chu et al. [35]	82.0	0.017	6.39 × 10^7^	244.00
Biffi et al. [36]	80.0	0.038	**3.83 × 10^6^**	15.00
Sun et al. [37]	81.7	**0.013**	7.25 × 10^6^	14.00
The proposed YOLOv5-v1	**86.8**	0.015	6.52 × 10^6^	**12.70**

**Figure A1:**
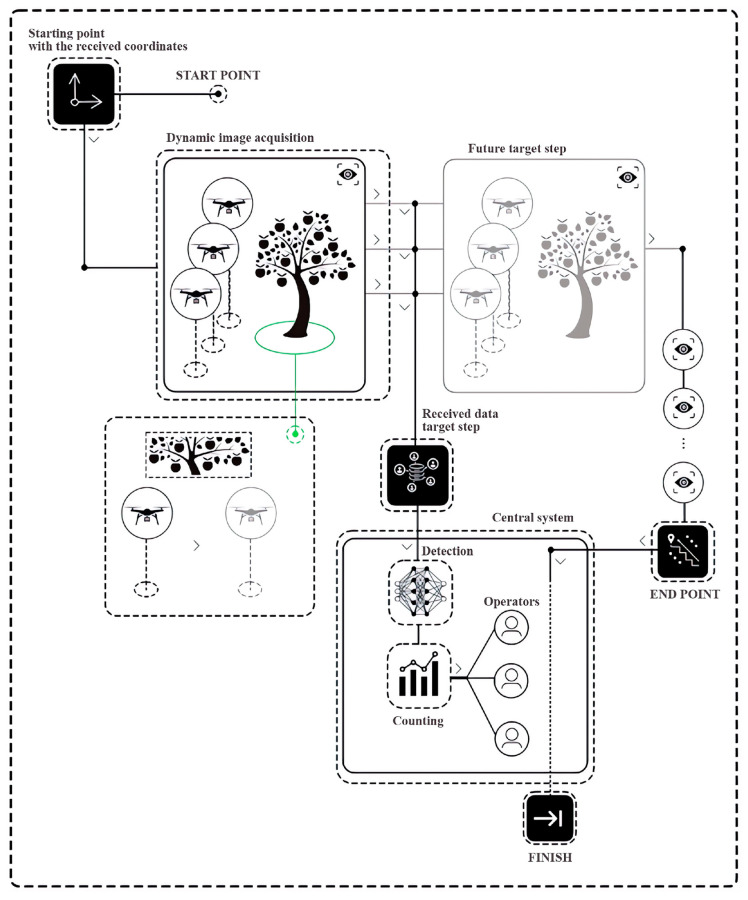
A systematic procedure for orchard monitoring using multiple UAVs. Commencing at a defined starting point with received coordinates, UAVs perform dynamic image acquisition. Sequentially, the images are transmitted to the central system for object detection and operator analysis, which includes the counting of identifiable items. Upon completion, the UAVs proceed to a future target, as determined by the received data. This workflow culminates at the end point, marking the end of a data collection cycle.

**Figure A2:**
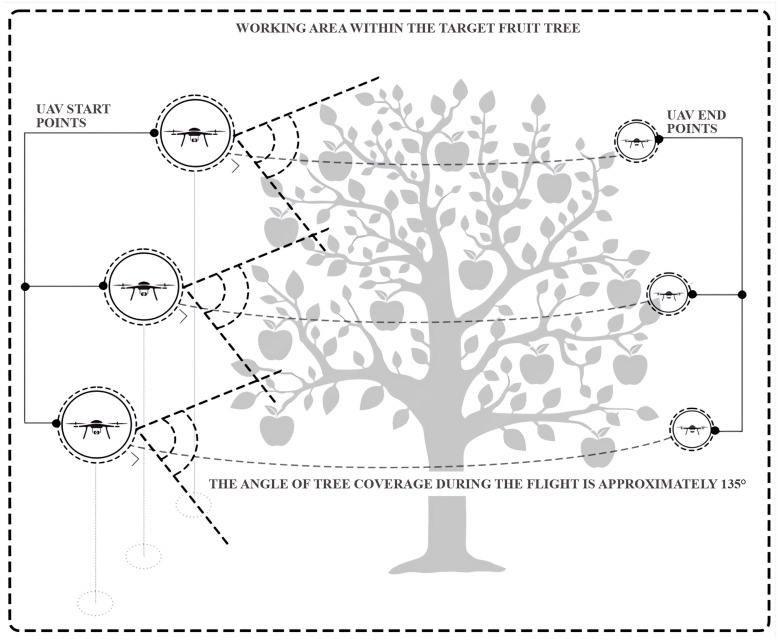
The scheme of the UAV group operation in the work zone around the target fruit tree. The UAVs start from multiple positions, converging in a coordinated pattern over a tree. The flight paths are designed to cover an angle of approximately 135 degrees around the tree, ensuring comprehensive visual data collection of the target fruit. This configuration allows for maximum spatial coverage, which is crucial for accurate and thorough structural object detection.

**Figure A3:**
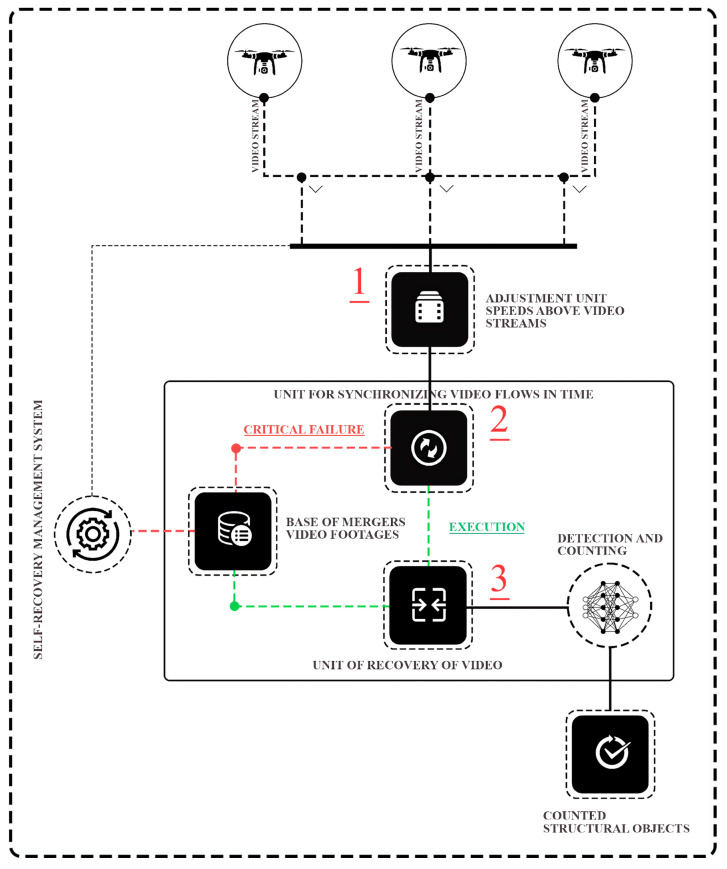
The schematic representation of an autonomous video management system of UAVs that highlights the synchronization of video streams, failure contingencies, and data recovery mechanisms. The system captures video streams from multiple UAVs, synchronizes them via an adjustment unit, and processes them for object detection and counting. The outcome is a consolidated count of structural objects, which is essential for precision agriculture analytics. Three main blocks of the system are marked with red numbers.

**Figure A4:**
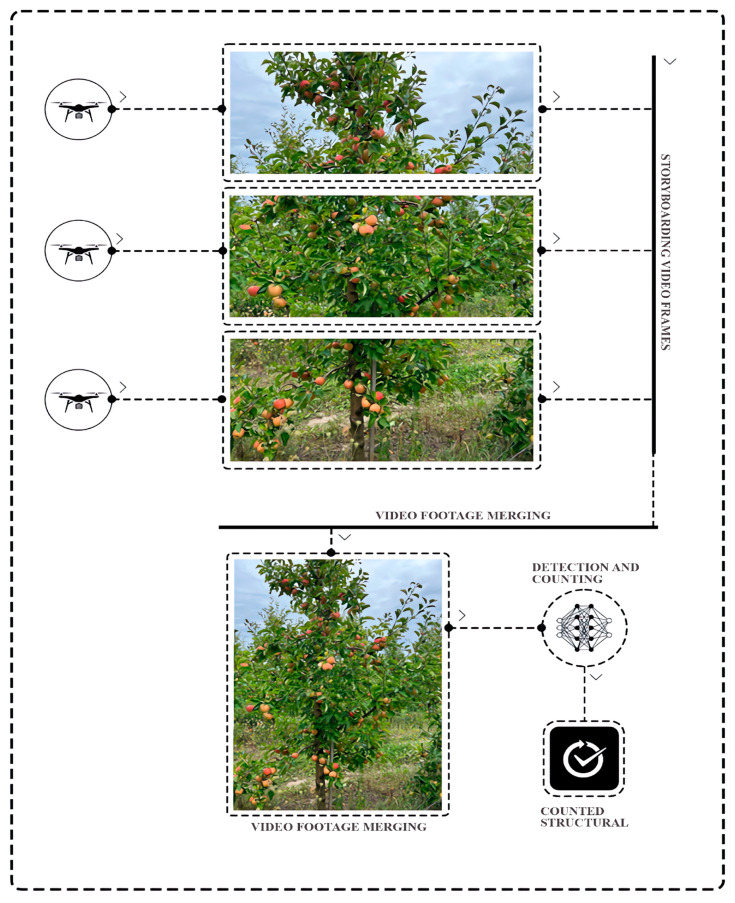
The scheme of the video frame merging process. Here, UAVs capture multiple perspectives of a target tree, which are then storyboarded into video frames for comprehensive visual coverage. These frames undergo merging to create a contiguous visual dataset, followed by the automated detection and counting of structural objects, i.e., fruits on the tree. The process flow encapsulates the critical steps from image acquisition to data synthesis, ending with counting structural objects.

## Data Availability

The experimental data used to support the findings of this study are available from the corresponding author upon request.

## Abbreviations

The following abbreviations included in the text are reported alphabetically:

AI	Artificial Intelligence
ATSS	Adaptive Training Sample Selection
CNN	Convolutional Neural Network
CSP	Cross Stage Partial
DCNN	Deep Convolutional Neural Network
DL	Deep Learning
FN	False Negative
FNR	False Negative Rate
FP	False Positive
FPR	False Positive Rate
FPN	Feature Pyramid Network
GNSS	Global Navigation Satellite System
GPS	Global Positioning System
IOU	Intersection Over Union
ISO	International Organization for Standardization
ML	Machine Learning
PSNR	Peak Signal-To-Noise Ratio
RGB	Red, Green, Blue
RTK	Real-Time Kinematic
SSIM	Structural Similarity Index Measure
TP	True Positive
TN	True Negative
UAV	Unmanned Aerial Vehicle
YOLO	You Only Look Once

## Appendix A

Appendix A describes in detail the method for dynamically capturing images of structural objects of a similar nature in a three-dimensional space. The method requires the step-by-step execution of several blocks that are presented below.

Block 1: UAV group take-off to the first starting point.

Step 1.1. This block begins at the start of the predefined mission. The operational area for the group of UAVs, as defined by software, includes specific sub-areas determined by the initial and final points located within the perimeters of the designated fruit tree.

The trajectory of the UAVs within the three-dimensional space is adjusted by modifying their positions within the software’s coordinate system. The fleet of UAVs then navigates toward the nearest fruit tree located in the current operational area. A graphical depiction of this type of flight is illustrated in Figure A1.

The verification of each UAV’s location within the software is a crucial step. It ensures that the physical positions of all UAVs in the fleet align with the predetermined software coordinates.

Step 1.2. The data gathered in step 1.1 is fed into the central module and all its subsystems. This process facilitates determining the operational statuses of all the system modules.

Block 2. Compilation of video data by the UAV fleet in the target area.

Step 2.1. Upon reaching the specified fruit tree, found according to the software’s coordinates, the UAV fleet generates an informational signal that is sent to the mission’s software. A critical procedure here is receiving a confirmation from the software system to turn on the video cameras of each UAV in the fleet. The extent of the area captured in the video stream is defined by the camera’s field of view on each UAV and any positional changes of the UAVs from the start to the end point, all located within the confines of the target fruit tree as defined in the software’s coordinate system.

Step 2.2. Each UAV, with its camera now activated, begins transmitting a video stream to the central system.

Step 2.3. The central system then processes the video data, and the information from step 2.2 is archived in the video stream database.

Step 2.4. Upon reaching the end point at the target fruit tree, the UAV group coordination software sends a signal to turn off the cameras on all UAVs.

Step 2.5. The mission management subsystem then confirms the deactivation of the cameras on each UAV and proceeds to physically power them down.

Step 2.6. Following this process, an analysis of the survey results is conducted. The system determines the operational statuses necessary for transitioning to the next fruit tree target.

Decisions regarding the ongoing operation of a UAV group, verification of the software mission’s integrity, and evaluation of the criticality level for the UAV group’s continued functioning are made by assessing the statuses of the software modules after completing Block 2.

The output of this method is a video stream that captures target objects within the fruit orchard.

The procedure for generating video streams targeting a specific fruit tree is meticulously designed and encoded within a software subsystem. This software subsystem, which is tasked with dynamically acquiring images of the designated structural objects, operates based on a thorough integration of distribution requirements, a multi-tiered hierarchy, and automation. This ensures that the UAV group autonomously navigates from the starting to the ending points as defined by the software mission. Given that the task of generating data for counting these structural objects is intricate and demanding, this subsystem outlines and codifies the following distinct characteristics:

- Programmatic coordinate values for placement;
- Programmatic coordinate values for starting and ending points;
- Duration at the start and end points;
- Number of detected structural objects;
- Actual number of structural objects;
- Type and characteristics of the video camera forming the video stream.

The data gathered from Block 2 informs the status and effectiveness of the UAV group’s operations within the active zone. If the software modules have successfully collected all the required attributes for continuing the mission, the subsystem overseeing the UAV group will issue a command to proceed to the next designated fruit tree.

The acquisition of dynamic images within the working zone, specifically within the confines of the target fruit tree, is presumed to occur under optimal environmental conditions. As such, the spatial arrangement of the UAVs for mission execution around the fruit tree is established during the UAV group’s formation. A key aspect of their vertical positioning relates to the width of camera coverage, which may vary due to the inclusion of different UAV models within the group. Typically, the video camera’s field of view for targeting the fruit tree is set at around 135 degrees. Figure A2 visually depicts how the UAV group executes its software mission within the operational zone surrounding the target fruit tree.

While transitioning between different targeted work zones of fruit trees, the UAV cameras are temporarily switched off. This precaution is taken to ensure the integrity of the target video data. The mission is considered to be successfully completed within a work zone when the UAV group physically arrives at the end point of that specific segment. At this point, the system’s main module signals the UAV group management module to cease the video streaming process, marking the end of operations in that zone.

The proposed methodologies for handling video data from individual work zones are structured for the collective processing of multiple fruit trees. This process relies on control and route planning subsystems. The creation of a multi-tiered, distributed software interface, integrated into a centralized system, facilitates the management of the UAV group and allows for immediate interaction with the system’s central module. This innovative approach enables the dynamic generation of images, thereby capturing specific structural objects within the working zone and the confines of the target fruit tree.

## Appendix B

The operational procedure of this innovative method is illustrated in Figure A3.

Block 1: Video stream speed adjustment. As the UAV group undertakes its software-directed mission across various work zones, it generates video sequences that are transmitted to a software module designated for identifying and calculating the intended structural objects. These video sequences are received by the detection software module at differing intervals, due to factors such as network connectivity variability, the specific camera models used on the UAVs, and the UAVs’ traversal speed within the work zones, among others. In light of these variations in video stream movement within the operational environment, the speed adjustment function constructs a software framework. This framework integrates mechanisms for processing the speed of video stream transmissions and establishes capabilities for halting and retrieving video sequences. The transition of the video stream speed adjustment function into a “waiting” state ensures the simultaneous reception of video sequences from all UAVs. This software framework includes distinct characteristics for each UAV: (1) a unique identifier for the UAV, (2) the byte composition of the video frame, (3) the frame rate of the video sequence, (4) the timestamp of the video frame’s reception by the speed adjustment function from the UAV, and (5) the encoding format of the video frame. Upon completion, the speed adjustment function transitions to the “execution” state, forwarding the assembled set of software structures to the subsequent processing block.

Block 2. Video stream time synchronization. This block operates based on the quantity of software structure sets that it receives from the prior block, adjusting the transmission speeds of the video streams. Its operations result in one of two scenarios:

Step 2.1. If the count of the software structure sets received does not align with the total number of UAVs in operation, the synchronization block enters a critical state. Consequently, it initiates a request to retrieve the most recently stored data structure from a database block that keeps saved, merged video frames.

Step 2.2. Upon the successful synchronization of video streams, the block transitions into the “frame extraction from video sequences” state, yielding a collection of software structures. These structures serve as the input for the subsequent video frame merging block.

Block 3. Video frame merging. Initially, this block verifies that the creation times of the software data structure sets are consistent, ensuring the data’s integrity. Following this verification, the video frames undergo a series of transformations:

Step 3.1. Conversion and adjustment. The byte set representing the dissected video frame is transformed into a file. Software mechanisms then scrutinize the file’s metadata to identify its distinctive features, such as file type, dimensions, color space, resolution, and channel depth.

Step 3.1.1. A series of images derived from step 3.1 is subjected to an angle adjustment through an affine transformation, aligning their geometric features.

Step 3.1.2. The adjusted images are then forwarded to the software merging function.

Step 3.2. Image merging. The cumulative efforts from the previous steps culminate in the creation of a single, unified image.

Step 3.2.1. Given potential disparities in the exposure and depth of field across different frames, the merged image might exhibit graphical artifacts. To counter this, a specialized software filter is applied to smooth the transitions and maintain clarity at the edges.

Step 3.2.2. The filtered frames are then compressed using a software mechanism.

Step 3.2.3. This compression, which is also based on affine transformation, optimizes the image for structural object detection and computation, reducing its dimensions while preserving resolution and depth.

Step 3.2.4. The software merging function receives the optimized video frames for final merging.

Step 3.2.5. The merging process consolidates several video frames into a single, seamless image, represented as a matrix in the software data structure. Each matrix element corresponds to the color value of an individual pixel, collectively depicting a continuous view of the fruit tree.

Step 3.2.6. The newly formed data are stored in an internal database of merged video frames.

Finally, the matrix-based software data structure is sent to a dedicated software module for the detection and quantification of homogeneous structural objects.

The workflow of the video frame merging block is detailed in Figure A4.

If this block successfully completes the video frame merging process, it then yields an output in the form of a software data structure, represented as a color-coded matrix. The merging block has a built-in function that ensures system stability by storing the data in an internal database of merged video frames, even in cases of critical system failure.

It should be also noted that a key benefit of the proposed method, when compared to the existing approaches, is its capability to store data from previous software missions. This feature enables users to perform immediate comparisons between current outcomes and historical data, enhancing the system’s utility and performance.

## Appendix C

**Table A1:** An outline of the layers and hyperparameters of the YOLOv5-v1 neural network architecture. It details the configuration of various layers, including the number of filters, kernel sizes, strides, activation functions, and specialized parameters such as batch normalization and reduction ratios.

Layer Type	Number of Filters	Kernel Size	Stride	Activation Function	Special Parameters	Notes
Focus	64	3	1	Leaky ReLU	-	Initial layer to capture fine details
Conv	128	3	2	Leaky ReLU	Batch normalization: True	Standard convolutional layer
BottleneckCSP-2	128	1 for transition layers	-	Leaky ReLU	Number of bottlenecks: 4	CSP with 2 bottleneck layers
BottleneckCSP	-	1 for transition layers	-	Leaky ReLU	Number of bottlenecks: 4	Number of bottlenecks: 4 in the first, followed by 2
SELayer	-	-	-	-	Reduction ratio: 16	Squeeze-and-excitation layer
SPP	256	-	-	Leaky ReLU	Pool sizes: 5,9,13	Spatial pyramid pooling to aggregate features
Upsample	-	-	-	-	Scale factor: 2	Upsampling to merge feature maps
Concat	-	-	-	-	Axis: -1	Concatenate feature maps from different scales
Detect	-	-	-	-	Anchors: 3 sizes per scale	Detection head with anchors for bounding boxes

## References

[1] Wijerathna-Yapa A., Pathirana R. (2022). Sustainable agro-food systems for addressing climate change and food security. Agriculture.

[2] Lee C.-C., Zeng M., Luo K. (2024). How does climate change affect food security? Evidence from China. Environ. Impact Assess. Rev..

[3] Kanike U.K. (2023). Factors disrupting supply chain management in manufacturing industries. J. Supply Chain. Manag. Sci..

[4] Buka S., Tkachuk V., Kondratiuk V., Tonkha O., Slobodyanyuk N. (2023). Prospects for agribusiness in Ukraine over the next 5 years. Int. J. Environ. Stud..

[5] Kalyta O., Barmak O., Radiuk P., Krak I. (2023). Facial emotion recognition for photo and video surveillance based on machine learning and visual analytics. Appl. Sci..

[6] Medvedeva Y., Kucher A., Lipsa J., Hełdak M. (2021). Human health risk assessment on the consumption of apples growing in urbanized areas: Case of Kharkiv, Ukraine. Int. J. Environ. Res. Public Health.

[7] Outhwaite C.L., McCann P., Newbold T. (2022). Agriculture and climate change are reshaping insect biodiversity worldwide. Nature.

[8] Xu J., Gu B., Tian G. (2022). Review of agricultural IoT technology. Artif. Intell. Agric..

[9] Reddy Maddikunta P.K., Hakak S., Alazab M., Bhattacharya S., Gadekallu T.R., Khan W.Z., Pham Q.-V. (2021). Unmanned aerial vehicles in smart agriculture: Applications, requirements, and challenges. IEEE Sens. J..

[10] Radiuk P., Barmak O., Krak I. (2021). An approach to early diagnosis of pneumonia on individual radiographs based on the CNN information technology. Open Bioinform. J..

[11] Sardar P., Ema R.R., Kabir S.S., Adnan M.N., Galib S.M. (2023). Severity stage identification and pest detection of tomato disease using deep learning. Int. J. Comput..

[12] Villacrés J., Viscaino M., Delpiano J., Vougioukas S., Auat Cheein F. (2023). Apple orchard production estimation using deep learning strategies: A comparison of tracking-by-detection algorithms. Comput. Electron. Agric..

[13] Tsouros D.C., Triantafyllou A., Bibi S., Sarigannidis P.G. Data acquisition and analysis methods in UAV-based applications for precision agriculture. Proceedings of the 2019 15th International Conference on Distributed Computing in Sensor Systems (DCOSS).

[14] Popescu D., Stoican F., Stamatescu G., Ichim L., Dragana C. (2020). Advanced UAV–WSN system for intelligent monitoring in precision agriculture. Sensors.

[15] Degieter M., De Steur H., Tran D., Gellynck X., Schouteten J.J. (2023). Farmers’ acceptance of robotics and unmanned aerial vehicles: A systematic review. Agron. J..

[16] Sachenko A., Kochan V., Turchenko V. (2003). Instrumentation for gathering data [DAQ Systems]. IEEE Instrum. Meas. Mag..

[17] Zhang C., Valente J., Kooistra L., Guo L., Wang W. (2021). Orchard management with small unmanned aerial vehicles: A survey of sensing and analysis approaches. Precis. Agric..

[18] Rizzo M., Marcuzzo M., Zangari A., Gasparetto A., Albarelli A. (2023). Fruit ripeness classification: A survey. Artif. Intell. Agric..

[19] Naranjo-Torres J., Mora M., Hernández-García R., Barrientos R.J., Fredes C., Valenzuela A. (2020). A review of convolutional neural network applied to fruit image processing. Appl. Sci..

[20] Sun Y., Fesenko H., Kharchenko V., Zhong L., Kliushnikov I., Illiashenko O., Morozova O., Sachenko A. (2022). UAV and IoT-based systems for the monitoring of industrial facilities using digital twins: Methodology, reliability models, and application. Sensors.

[21] Awais M., Li W., Cheema M.J.M., Zaman Q.U., Shaheen A., Aslam B., Zhu W., Ajmal M., Faheem M., Hussain S. (2023). UAV-based remote sensing in plant stress imagine using high-resolution thermal sensor for digital agriculture practices: A meta-review. Int. J. Environ. Sci. Technol..

[22] Lambertini A., Mandanici E., Tini M.A., Vittuari L. (2022). Technical challenges for multi-temporal and multi-sensor image processing surveyed by UAV for mapping and monitoring in precision agriculture. Remote Sens..

[23] Skorobogatov G., Barrado C., Salamí E. (2020). Multiple UAV systems: A survey. Un. Sys..

[24] Shi K., Zhang X., Xia S. (2020). Multiple swarm fruit fly optimization algorithm based path planning method for multi-UAVs. Appl. Sci..

[25] Khan S., Tufail M., Khan M.T., Khan Z.A., Iqbal J., Wasim A. (2022). A novel framework for multiple ground target detection, recognition and inspection in precision agriculture applications using a UAV. Un. Sys..

[26] Su J., Zhu X., Li S., Chen W.-H. (2023). AI meets UAVs: A survey on AI empowered UAV perception systems for precision agriculture. Neurocomputing.

[27] Li H., Xie X., Du P., Xi J. Cooperative object recognition method of multi-UAVs based on decision fusion. Proceedings of the 2021 33rd Chinese Control and Decision Conference (CCDC).

[28] Chen R., Zhang C., Xu B., Zhu Y., Zhao F., Han S., Yang G., Yang H. (2022). Predicting individual apple tree yield using UAV multi-source remote sensing data and ensemble learning. Comput. Electron. Agric..

[29] Bate J.R.R.T. (2022). Applying Deep Learning to Estimate Fruit Yield in Agriculture 4.0 Systems. Master’s Thesis.

[30] Wang C., Liu S., Wang Y., Xiong J., Zhang Z., Zhao B., Luo L., Lin G., He P. (2022). Application of convolutional neural network-based detection methods in fresh fruit production: A comprehensive review. Front. Plant Sci..

[31] Mao D., Sun H., Li X., Yu X., Wu J., Zhang Q. (2023). Real-time fruit detection using deep neural networks on CPU (RTFD): An edge AI application. Comput. Electron. Agric..

[32] Bodyanskiy Y., Deineko A., Skorik V., Brodetskyi F. (2022). Deep neural network with adaptive parametric rectified linear units and its fast learning. Int. J. Comput..

[33] Tu S., Pang J., Liu H., Zhuang N., Chen Y., Zheng C., Wan H., Xue Y. (2020). Passion fruit detection and counting based on multiple scale Faster R-CNN using RGB-D Images. Precis. Agric..

[34] Mai X., Zhang H., Jia X., Meng M.Q.-H. (2020). Faster R-CNN with classifier fusion for automatic detection of small fruits. IEEE Trans. Autom. Sci. Eng..

[35] Chu P., Li Z., Lammers K., Lu R., Liu X. (2021). Deep learning-based apple detection using a suppression Mask R-CNN. Pattern Recognit. Lett..

[36] Biffi L.J., Mitishita E., Liesenberg V., dos Santos A.A., Gonçalves D.N., Estrabis N.V., de Silva J.A., Osco L.P., Ramos A.P.M., Centeno J.A.S. (2021). ATSS deep learning-based approach to detect apple fruits. Remote Sens..

[37] Sun Y., Zhang D., Guo X., Yang H. (2023). Lightweight algorithm for apple detection based on an improved YOLOv5 model. Plants.

[38] Zheng Z., Xiong J., Wang X., Li Z., Huang Q., Chen H., Han Y. (2023). An efficient online citrus counting system for large-scale unstructured orchards based on the unmanned aerial vehicle. J. Field Robot..

[39] Welch G.F. (2020). Kalman filter. Computer Vision: A Reference Guide.

[40] Gabrovšek B., Novak T., Povh J., Rupnik Poklukar D., Žerovnik J. (2020). Multiple Hungarian method for K-assignment problem. Mathematics.

[41] Ghorbani H. (2019). Mahalanobis distance and its application for detecting multivariate outliers. Facta Univ. Ser. Math. Inform..

[42] (2019). Photography—Digital still Cameras—Determination of Exposure Index, ISO Speed Ratings, Standard Output Sensitivity, and Recommended Exposure Index.

[43] Grandini M., Bagli E., Visani G. (2020). Metrics for multi-class classification: An overview. arXiv.

[44] Dempsey P. (2021). Reviews consumer technology: The teardown: Apple IPhone Pro 13 smartphone. Eng. Technol..

[45] Langer P., Fleisch E., Barata F. (2023). CLAID: Closing the loop on AI & data collection—A cross-platform transparent computing middleware framework for smart edge-cloud and digital biomarker applications. arXiv.

[46] Mishra A. (2019). Amazon Sagemaker. Machine Learning in the AWS Cloud: Add Intelligence to Applications with Amazon Sagemaker and Amazon Rekognition.

[47] Paszke A., Gross S., Massa F., Lerer A., Bradbury J., Chanan G., Killeen T., Lin Z., Gimelshein N., Antiga L., Wallach H., Larochelle H., Beygelzimer A., Alché-Buc F., Fox E., Garnett R. (2019). PyTorch: An imperative style, high-performance deep learning library. Proceedings of the Advances in Neural Information Processing Systems (NeurIPS 2019).

[48] Al Ghadani A.K.A., Mateen W., Ramaswamy R.G., Maglogiannis I., Iliadis L., Pimenidis E. (2020). Tensor-based CUDA optimization for ANN inferencing using parallel acceleration on embedded GPU. Artificial Intelligence Applications and Innovations.

[49] Gollapudi S., Gollapudi S. (2019). OpenCV with Python. Learn Computer Vision Using OpenCV: With Deep Learning CNNs and RNNs.

[50] Horé A., Ziou D. Image quality metrics: PSNR vs. SSIM. Proceedings of the 2010 20th International Conference on Pattern Recognition (ICPR-2010).

[51] Melnychenko O., Savenko O., Radiuk P. Apple detection with occlusions using modified YOLOv5-v1. Proceedings of the 12th IEEE International Conference on Intelligent Data Acquisition and Advanced Computing Systems: Technology and Applications (IDAACS’2023).

